# Enantioseparation, quantification, molecular docking and molecular dynamics study of five β-adrenergic blockers on Lux-Cellulose-2 column

**DOI:** 10.1186/s13065-023-00925-2

**Published:** 2023-03-16

**Authors:** Ola Ahmed Saleh, Amr Mohamed Badawey, Hassan Y. Aboul-Enein, Marwa Ahmed Fouad

**Affiliations:** 1 Medicinal and Pharmaceutical Chemistry Department, Pharmaceutical and Drug Industries Research Division, National Research Centre (ID: 60014618), P.O. Box 12622, Giza, Egypt; 2 Analytical Chemistry Department, Faculty of Pharmacy, Cairo University, Kasr El-Aini St., Cairo, 11562 Egypt; 3 Pharmaceutical Chemistry Department, Faculty of Pharmacy, Cairo University, Kasr El-Aini St., Cairo, 11562 Egypt; 4 Pharmaceutical Chemistry Department, School of Pharmacy, NewGiza University, NewGiza, Km 22 Cairo-Alexandria Desert Road, Cairo, Egypt

**Keywords:** Lux-Cellulose-2 chiral column, Enantioseparation, β-adrenergic blockers, HPLC, Molecular docking, Molecular dynamics

## Abstract

Enantioseparation of five β-adrenergic blockers was studied using two mobile phases on a cellulose tris(3‐chloro‐4‐methylphenylcarbamate) (Lux-Cellulose-2) chiral column in normal phase mode. The first mobile phase composed of n-hexane: ethanol: diethylamine 60: 40: 0.1 by volume has successfully resolved the chromatographic peaks of three pairs of β-adrenergic blockers namely, bisoprolol, carvedilol and atenolol. A mixture of n-hexane: ethanol: diethyl amine 75: 25: 0.1 by volume was used as the second mobile phase to separate the four pairs of enantiomers, metoprolol, carvedilol, nebivolol and atenolol with high resolution values. The mobile phases were pumped at a flow rate 1 mL/min with column temperature 25 °C using a UV detector at 230 nm. Molecular docking simulations of the five pairs of enantiomers was carried out in the cavities of the chiral stationary phase to gain a better understanding of the interaction between analyte enantiomers and chiral stationary phase and to better understand the mechanism of chiral recognition. According to the results, hydrogen bond interactions and π-π- interactions were the main types of interaction involved in the chiral recognition. Molecular dynamics simulation was performed to investigate the solvent effect on the interaction of the five pair of enantiomers in the chiral stationary phase cavity under dynamic conditions.

**Supplementary Information:**

The online version contains supplementary material available at 10.1186/s13065-023-00925-2.

## Introduction

In 1848, Louis Pasteur separated two isomers of sodium ammonium tartrate. Thereby, the concept of separation of isomers was established and further studied [1]. Due to the biological significance of chirality [2], the analysis of chiral compounds becomes increasingly important in many fields such as food, agrochemical and pharmaceutical fields [3–8] and various techniques have been developed for their determination [9]. High performance liquid chromatography (HPLC) is considered to be the most effective and widely used technique for the separation of different enantiomers using chiral stationary phases (CSPs) [3, 5, 6, 8, 10–13].

**Fig. 1:**
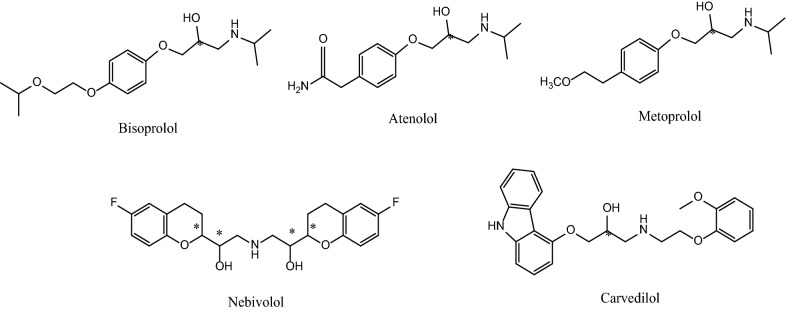
Chemical structure of β-adrenergic blockers. Asterisk (*) denotes the position of the chiral center

β-Adrenergic blockers such as bisoprolol, carvedilol, atenolol, metoprolol and nebivolol, Fig. 1, are chiral hydroxylamine containing compounds, which have been used for the treatment of hypertension, angina pectoris, cardiac arrhythmias, and glaucoma [14]. Usually, the (S)-enantiomers of these drugs show higher pharmacological activity due to their higher receptor affinities and good stereospecific fitting than their (R)-enantiomer [15–17].

Some biotransformation pathways of β-adrenergic blockers have stereospecificity in humans [18]. As a result, for a better understanding of β-adrenergic blockers, the method of their enantioseparation and chiral recognition mechanism should be studied. Some reports on the enatioseparation of β-adrenergic blockers by using HPLC with chiral stationary phase such as Chiralpak AD-H, Chiralpak IA, Chiralpak IB and Chiralpak ID were published. Where the enantiomeric separation of β-adrenergic blockers was a result of more than one type of interaction between solutes and CSP but mainly hydrogen bonding interaction. Besides hydrogen bonding, there were other types of interaction that was independent on solvent polarity. The presence of both groups close to the chiral centers and substituent groups on the phenyl rings which were far from the chiral centers may contribute for the good separation [19–22].

Molecular docking has been widely developed in many fields, one of them is the simulation study of the interactions between enantiomers and the chiral selector to determine the mechanism for chiral separation [23, 24]. Recently, theoretical methods such as molecular dynamics (MD) are widely used to study the inclusion complexes of the chiral stationary phases with enantiomers to better understand the chiral resolution mechanism [25, 26].

In this work, the investigation of enantioselective separation, quantification and chiral `recognition of five β‐adrenergic blockers namely bisoprolol, carvedilol, atenolol, metoprolol and nebivolol (Fig. 1) on a cellulose tris(3‐chloro‐4‐methyl phenyl carbamate (Lux-Cellulose-2) column with Molecular Operating Environment (MOE) molecular docking and molecular dynamics studies is reported. The five studied β‐adrenergic blockers have not been reported to be enantiomerically separated using Lux-Cellulose 2 column. Also, no molecular simulation studies were reported describing the interaction of the analytes under investigation and the selected chiral stationary phase. First, chiral separation of the analytes was performed by HPLC. In order to achieve our goal, the mobile phase composition was optimized by varying the type and concentration of additives and by changing the ratio of n-hexane and ethanol with 0.1% diethylamine. Second, to explain the chiral differentiation mechanism, molecular docking of each enantiomer in the chiral stationary phase Lux cellulose-2 was carried out. The enantioseparation achieved by HPLC was explained by the obtained pose of the most stable complex for each enantiomer with visualization of the intermolecular interactions responsible for separation. The difference in binding energy scores predicted for each pair of enantiomers was qualitatively consistent with the enantiomeric resolution in HPLC analysis. Molecular dynamics study was carried out to investigate the role of mobile phase solvent molecules.

## Materials and methods

### Chemicals and reagents

The standard racemic substances of bisoprolol, carvedilol, atenolol, metoprolol and nebivolol, were obtained from Sigma- Aldrich, Germany. Nebivolol is a racemate composed of d-Nebivolol and l-Nebivolol with the stereochemical designations of [SRRR]-nebivolol and [RSSS]-nebivolol, respectively. HPLC-grade n-hexane, ethanol and methanol were purchased from Sigma- Aldrich, Germany. Diethylamine (DEA) was of analytical reagent grade and supplied from Sigma- Aldrich, Germany.

### Equipment

The enantioseparation was performed on an Agilent HPLC unit; 1100 series apparatus; equipped with a quaternary pump, a vacuum degasser, a column oven and a diode array UV detector. The used chiral column was Lux‐Cellulose‐2 (cellulose tris(3‐chloro‐4‐methyl phenyl carbamate)) (250 × 4.6 mm, 3 µm particle size) purchased from Phenomenex (Torrance, USA). Chromatographic data acquisition and analysis was performed by Hewlett-Packard Chemstation software for LC 3D systems; Rev. B.03.01 (317) Copyright© Agilent Technologies 2001–2007. All the molecular docking studies were carried out using Molecular Operating Environment (MOE, 2020.0901) software.

### Chromatographic conditions

Two analytical methods were developed and accordingly different combinations of n-hexane: ethanol: diethylamine were prepared as the mobile phase using a flow rate of 1.0 mL/min. In the first method, Mobile phase A, composed of n-hexane: ethanol: diethylamine, 60: 40: 0.1, by volume, was used for the enantioseparation of bisoprolol, carvedilol and atenolol. In the second method, Mobile phase B composed of n-hexane: ethanol: diethylamine 75: 25: 0.1, by volume, was used for the enantioseparation of metoprolol, carvedilol, nebivolol and atenolol. The mobile phases were filtered and degassed daily before use. The separation was carried out at 25 °C. The detection wavelength was fixed at 230 nm. The retention factors (k), separation factors ($\mathrm{\alpha }$) and resolution factors (Rs) were calculated.

### Preparation of stock and working solutions

The stock solutions of bisoprolol, atenolol, metoprolol and nebivolol (1 mg/mL) and carvedilol (0.4 mg/mL) were prepared in methanol. Working solutions of bisoprolol and atenolol were prepared in a concentration range from 50 to 300 $\mathrm{\mu g}/\mathrm{mL}$ and carvedilol 20–120 $\mathrm{\mu g}/\mathrm{mL}$ in methanol. In the second method, atenolol, metoprolol and nebivolol were diluted to 50–250 $\mathrm{\mu g}/\mathrm{mL}$ and carvedilol to 20–100 $\mathrm{\mu g}/\mathrm{mL}$ with methanol. All solutions were filtered through a nylon membrane of 0.45 m$\upmu$ pore size. The prepared solutions were then filtered through nylon membrane of 0.45 µm pore size before being injected into the HPLC system with an injection volume of 5 µL.

### Calculation

The chromatographic parameters of retention factor (k), separation factor ($\propto )$ and resolution (Rs) were calculated as follows: k = (t_R_ − t_0_)/t_o_, where t_R_ and t_0_ are the retention times of the analyte and unretained solute, respectively, $\propto$=k_2_/k_1_, where k_1_ and k_2_ are the retention factors of the first and second eluted enantiomers; and R_5_ = 2(t_2_ − t_1_)/(w_1_ + w_2_), where t_1_ and t_2_ were the retention times of the successively eluted enantiomers, and w_1_ and w_2_ are the peak widths of the first and second eluted enantiomers, respectively.

### Molecular docking simulation study

The conformations of the stereoisomers (as ligands) and Lux Cellulose-2 stationary phase (as receptor) were sketched, energy minimized, and all the molecular docking studies were carried out using Molecular Operating Environment (MOE, 2020.0901) software. Energy minimization was performed until a root mean square deviation (RMSD) gradient of 0.05 kcal/ mol Å with Amber10: EHT force field and then partial charges were automatically calculated. All CSP atoms were defined as the docking site. Triangle Matcher placement method and London dG scoring function were used for the docking protocol and induced fit placement and GBVI/WSA dG scoring function were used for the refinement of the produced poses using Amber10: EHT force field.

### Molecular dynamics simulation study

In order to study the stability of the enantiomer-CSP complex, a molecular dynamic simulation study was performed. The best pose of enantiomers in the CSP cavity obtained from the docking study was retrieved and saved in MOE format to be used as initial structure for MD simulation. The whole enantiomer-CSP complex system was submitted to molecular dynamic simulation over 600 picoseconds (ps) period. All hydrogens were added, partial charges have been calculated and the energy of the molecular system was minimized to RMS gradient of 1.0. The inclusion complex of CSP with each enantiomer was surrounded by ethanol molecules as the solvent. The NPA was used to study the molecular dynamics of ligands and MMFF94x was chosen as a force field. All electrostatics, restraints, and Van der Waals forces were allowed. The Simulation process was conducted for 600 ps including 100 ps for equilibrium and 500 ps for production.

## Results and discussion

Enantioseparation and quantification of five β-adrenergic blockers namely, bisoprolol, carvedilol, atenolol, metoprolol and nebivolol (Fig. 1), was performed by HPLC using a chiral stationary phase, Lux-Cellulose-2 column. In order to achieve the goal, the mobile phase composition was optimized by varying the concentration of alcohol modifier and basic additive.

For the investigation of the chiral recognition of the five β-adrenergic blockers with the chiral stationary phase, molecular docking and dynamic studies using Molecular Operating Environment (MOE, 2020.0901) software were performed.

### Method optimization

#### Effect of the type and concentration of additives on enantioselectivity

The analytes were enantiomerically separated using n-hexane: ethanol system without additive at first. However, it was found that the peaks were rather broad and showed severe tailing in some chromatographic results. The effect of mobile phase additives was investigated for a variety of compounds under subcritical and supercritical conditions using packed columns. The temperature of the mobile phase is the major factor in the extent of this dependence [27].

In our preliminary study, 0.1% diethylamine, 0.1% triethylamine or 0.1% methylethylamine was added to the mobile phase, generally, this was found to improve the peak shapes. Comparing the enantioseparation results with different additives, almost all the analytes were better separated with higher $\propto$ and Rs values when 0.1% diethylamine was used as an additive.

#### Effect of changing the ratio of n-hexane and ethanol with 0.1% diethylamine

**Fig. 2:**
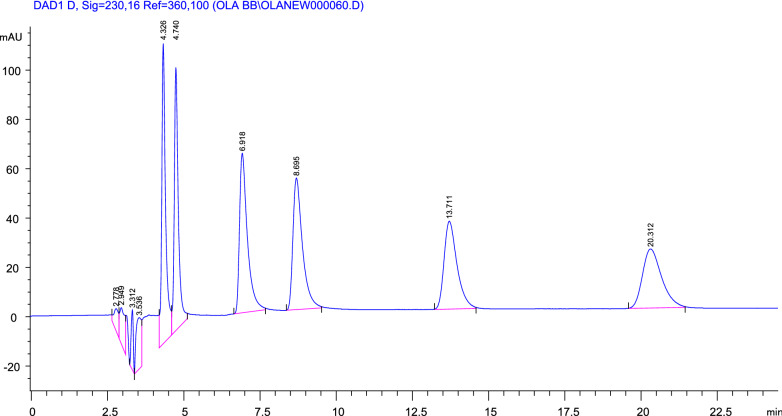
High performance liquid chromatography (HPLC) chromatograms of chiral separation of three β-adrenergic blockers on Lux-Cellulose-2 at a flow rate of 1.0 mL/min with column temperature of 25 °C. The detection wavelength was 230 nm. Mobile phase n-hexane: ethanol: DEA (60:40 0.1 by volume). Retention times of Bisoprolol enantiomers 250 µg/mL are 4.33 min, 4.74 min. Retention times of carvedilol enantiomers 100 µg/mL are 6.92 min, 8.70 min. Retention times of atenolol enantiomers 250 µg/mL are 13.71 min, 20.31 min

Different ratios of n-hexane: ethanol: 0.1% diethylamine were examined. A mixture of n-hexane: ethanol: diethylamine (60: 40: 0.1 by volume) was used for enantiomeric separation and quantification of three β-adrenergic blockers namely, bisoprolol, carvedilol and atenolol with retention time 4.33, 4.74, with column resolution 2.17 for bisoprolol; 6.92, 8.70, with column resolution 3.73 for carvedilol and 13.71, 20.31, with column resolution 7.04 for atenolol as shown in (Fig. 2, Table 1).

**Table 1:** System suitability parameters of the proposed HPLC method for quantitative determination of bisoprolol, carvedilol and atenolol enantiomers using mobile phase n-hexane: ethanol: DEA (60: 40: 0.1 by volume)

Parameter	Bisoprolol		Carvedilol		Atenolol	
	Enant. 1	Enant.2	Enant.1	Enant.2	Enant.1	Enant.2
Retention time (R_t_)(min)	4.33	4.74	6.92	8.70	13.71	20.31
Selectivity ($\propto$)	1.23	1.10	1.46	1.26	1.57	1.47
Resolution (R_s_)	2.17	2.17	3.73	3.73	7.04	7.04
Number of theoretical plates (N)	7531	7595	3924	4088	5221	5042
Height equivalent to theoretical plates (mm)	121.76	106.58	64.71	53.31	35.55	23.93

**Fig. 3:**
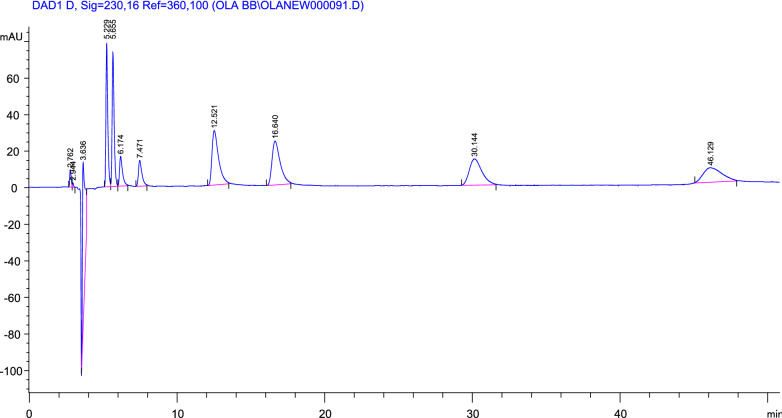
High performance liquid chromatography (HPLC) chromatogram of enantioseparation of four β–adrenergic blockers on Lux-Cellulose-2 at flow rate of 1.0 mL/min with the column temperature of 25 °C. The detection wavelength was 230 nm. Mobile phase n-hexane: ethanol: DEA (75: 25: 0.1 by volume). Retention times of metoprolol enantiomers 220 µg/mL are 5.23 min, 5.66 min. Retention times of nebivolol enantiomers 220 µg/mL are 6.17 min, 7.47 min. Retention times of carvedilol enantiomers 88 µg/mL are 12.52 min, 16.64 min. Retention times of atenolol enantiomers 220 µg/mL are 30.14 min, 46.13 min

While a mixture of n-hexane: ethanol: diethylamine (75: 25: 0.1 by volume) was used for enantioseparation and quantification of four β-adrenergic blockers, metoprolol, nebivolol, carvedilol and atenolol with retention time values 5.23, 5.66, with column resolution 1.94 for metoprolol; 6.17, 7.47, with column resolution 3.35 for nebivolol; 12.52, 16.64, with column resolution 4.57 for carvedilol and 30.14, 46.13, with column resolution 8.08 for atenolol as shown in (Fig. 3, Table 2).

**Table 2:** System suitability parameters of the proposed HPLC method for quantitative determination of metoprolol, nebivolol carvedilol and atenolol enantiomers using mobile phase n-hexane: ethanol: DEA (75: 25: 0.1 by volume)

Parameter	Metoprolol		Nebivolol		Carvedilol		Atenolol	
	Enant. 1	Enant.2	Enant. 1	Enant.2	Enant. 1	Enant.2	Enant. 1	Enant.2
Retention time (R_t_) (min)	5.23	5.66	6.17	7.47	12.52	16.64	30.14	46.13
Selectivity ($\propto$)	1.45	1.09	1.08	1.21	1.70	1.34	1.80	1.54
Resolution (R_s_)	1.94	1.94	3.35	3.35	4.57	4.57	8.08	8.08
Tailing factor	0.62	0.64	0.51	0.55	0.50	0.57	0.68	0.65
Number of theoretical plates (N)	9082	8888	5104	5176	4044	4456	5948	5430
Height equivalent to theoretical plates (mm)	78.41	73.31	16.28	14.06	29.82	23.97	14.35	7.92

#### Construction of calibration curve

Different aliquots (0.5–3.0 mL) of bisoprolol (1 mg/mL), carvedilol (0.4 mg/mL) and atenolol (1 mg/mL) were accurately transferred into a series of 10 mL volumetric flasks and the volume was then completed to the mark with methanol in case of using mobile phase A while in case of using mobile phase B, aliquots of 0.5–2.5 mL of metoprolol (1 mg/mL), nebivolol (1 mg/mL), carvedilol (0.4 mg/mL), atenolol (1 mg/mL) were used. Five µL of these solutions were injected in triplicate into the HPLC system on Lux‐Cellulose‐2 (cellulose tris (3‐chloro‐4‐methylphenyl carbamate) (250 × 4.6 mm, 3 µm particle size). The chromatograms were recorded at 230 nm as shown in Figs. 2 and 3 and the calibration curve for each drug was plotted and the corresponding regression equation was calculated.

$$ {\text{Regression equation}}:{\text{Y}} = {\text{ Slope}}.{\text{ X }} \pm {\text{ Intercept}} $$

where Y is the area under the curve and X is the concentration µg/mL.

#### Method validation

The ICH guidelines were followed for analytical method validation [28].

##### Linearity

Calibration graph for each drug was constructed by plotting peak area at 230 nm versus the corresponding concentration. It was observed that a linear relationship was obtained. The linearity of the proposed method was evaluated by triplicate of each experiment. The calibration graph for each drug was constructed within the previously mentioned concentration range and the corresponding regression equation was computed.

##### Accuracy

The previously mentioned chromatographic conditions were used for the determination of different concentrations of each drug within the linearity range. The concentrations were calculated using the linear regression equation for each enantiomer as shown in Tables 3 and 4.

**Table 3:** Regression and validation parameters of the proposed HPLC method for quantitative determination of bisoprolol, carvedilol and atenolol enantiomers using mobile phase n-hexane: ethanol: DEA (60: 40: 0.1 by volume)

Parameters	Bisoprolol		Carvedilol		Atenolol	
	Enant. 1	Enant.2	Enant. 1	Enant.2	Enant. 1	Enant.2
Linearity						
Slope(Au-Sec.ml/µg)	3.2975	3.3319	11.8970	11.8030	4.0228	3.9870
Intercept (Au-Sec)	− 1.2267	− 1.1400	− 15.9670	− 9.9133	50.3600	51.4800
Correlation coefficient r	0.9995	0.9995	0.9996	0.9996	0.9995	0.9995
Range ($\upmu$g/mL)	50–300	50–300	20–120	20–120	50–300	50–300
Accuracy (Mean + R.S.D.%)	99.93 + 0.942	99.93 + 0.432	99.98 + 1.030	100.09 + 0.956	100.35 + 0.849	99.97 + 1.185
*Robustness (RSD %)	0.821	0.426	0.962	0.843	0.787	0.982
**Robustness (RSD %)	0.624	0.408	0.773	0.641	0.691	0.522
Precision (RSD %)						
Repeatabilty^a^	0.531	0.314	0.732	0.548	0.492	0.821
Reproducibility^b^	0.722	0.409	0.811	0.699	0.588	0.911
LOD^c^ ($\upmu$g/mL)	6.7	6.7	2.7	2.7	6.7	6.7
LOQ^c^ ($\upmu$g/mL)	20	20	8	8	20	20

^a^Intra-day (n = 3), average of three concentration of each drug repeated three times within the same day

^b^Inter-day (n = 3), average of three concentration of each drug repeated three times three consecutive days

^c^LOD = 3.3 (SD/S), LOQ = 10 (SD/S), where SD is the standard deviation of regression residuals and S is the slope of calibration curves

*The robustness of the proposed method was investigated by analysis of samples under deliberate change in flow rate ± 0.1 mL/min

**The robustness of the proposed method was investigated by analysis of samples under deliberate change in Temperature ± 0.1 °C

**Table 4:** Regression and validation parameters of the proposed HPLC method for quantitative determination of Metoprolol, Nebivolol, carvedilol and atenolol enantiomers using mobile phase n-hexane: ethanol: DEA (75: 25: 0.1by volume)

Parameters	Metoprolol		Nebivolol		Carvedilol		Atenolol	
	Enant.1	Enant.2	Enant.1	Enant.2	Enant.1	Enant.2	Enant.1	Enant.2
Linearity								
Slope (Au-Sec.ml/µg)	3.2903	3.3722	1.0659	1.1315	11.3710	11.4470	3.8646	3.6325
Intercept (Au-Sec)	− 3.3282	− 6.3580	− 4.4288	− 2.0739	− 13.5980	− 17.7210	17.4170	25.2190
Correlation coefficient r	0.9996	0.9995	0.9995	0.9997	0.9996	0.9996	0.9995	0.9995
Range ($\upmu$g/ml)	50–250	50–250	50–250	50–250	20–100	20–100	50–250	50–250
Accuracy (Mean + R.S.D.%)	99.96 + 1.022	100.21 + 0.867	99.90 + 0.587	100.40 + 0.898	100.29 + 1.088	99.94 + 1.000	100.29 + 0.898	100.12 + 1.405
*Robustness (RSD %)	0.996	0.853	0.531	0.832	0.992	0.997	0.845	0.998
**Robustness (RSD %)	0.931	0.792	0.622	0.774	0.832	0.851	0.641	0.826
Precision (RSD %)								
Repeatabilty^a^	0.821	0.612	0.481	0.765	0.851	0.805	0.733	0.914
Reproducibility^b^	0.933	0.693	0.531	0.801	0.942	0.912	0.799	0.982
LOD^c^ ($\upmu$g/mL)	6.7	6.7	6.7	6.7	2.7	2.7	6.7	6.7
LOQ^c^ ($\upmu$g/mL)	20	20	20	20	8	8	20	20

^a^Intra-day (n = 3), average of three concentrations of each drug, repeated three times within the same day

^b^Inter-day (n = 3), average of three concentrations of each drug repeated three times in three consecutive days

^c^LOD = 3.3 (SD/S), LOQ = 10(SD/S), where SD is the standard deviation of regression residuals and S is the slope of calibration curves

*The robustness of the proposed method was investigated by analysis of samples under deliberate change in flow rate ± 0.1 mL/min

**The robustness of the proposed method was investigated by analysis of samples under deliberate change in Temperature ± 0.1 °C

The recovery percentages, the mean recoveries and RSD values were then calculated as shown in Tables 5, 6, 7.

**Table 5:** Accuracy of the proposed HPLC method using mobile phase n-hexane: ethanol: DEA (60: 40: 0.1 by volume) for determination of bisoprolol, carvedilol and atenolol enantiomers

Bisoprolol					Carvedilol					Atenolol				
Taken $\mathrm{\mu g}/\mathrm{mL}$	Found $\mathrm{\mu g}/\mathrm{mL}$ enant.1	Recovery % enant.1	Found $\mathrm{\mu g}/\mathrm{mL}$ enant.2	Recovery % enant.2	Taken $\mathrm{\mu g}/\mathrm{mL}$	Found $\mathrm{\mu g}/\mathrm{mL}$ enant.1	Recovery % enant.1	Found $\mathrm{\mu g}/\mathrm{mL}$ enant.2	Recovery % enant.2	Taken $\mathrm{\mu g}/\mathrm{mL}$	Found $\mathrm{\mu g}/\mathrm{mL}$ enant.1	Recovery % enant.1	Found $\mathrm{\mu g}/\mathrm{mL}$ enant.2	Recovery % enant.2
50.00	49.80	99.60	49.86	99.72	20.00	20.30	101.50	20.06	100.30	50.00	50.65	101.30	49.66	99.32
100.00	98.93	99.38	99.38	99.38	40.00	40.16	100.40	40.27	100.68	100.00	100.61	100.61	101.52	101.52
150.00	152.00	101.33	150.41	100.27	60.00	59.27	98.78	59.84	99.73	150.00	149.34	99.56	150.23	100.15
200.00	198.66	99.33	199.12	99.56	80.00	79.18	98.98	78.79	98.49	200.00	198.86	99.43	196.65	98.33
250.00	250.45	100.18	251.16	100.46	100.00	99.71	99.71	100.01	100.01	250.00	249.67	99.87	248.57	99.43
300.00	300.60	100.20	300.47	100.16	120.00	120.63	100.53	121.57	101.31	300.00	303.98	101.33	303.18	101.06
Mean		99.93		99.93			99.98		100.09			100.35		99.97
R.S.D		0.942		0.432			1.030		0.956			0.849		1.185

**Table 6:** Accuracy of the proposed HPLC method using mobile phase n-hexane: ethanol: DEA (75: 25: 0.1 by volume) for quantitative determination of metoprolol and nebivolol enantiomers

Metoprolol					Nebivolol				
Taken $\mathrm{\mu g}/\mathrm{mL}$	Found $\mathrm{\mu g}/\mathrm{mL}$ enant.1	Recovery % enant.1	Found $\mathrm{\mu g}/\mathrm{mL}$ enant.2	Recovery % enant.2	Taken $\mathrm{\mu g}/\mathrm{mL}$	Found $\mathrm{\mu g}/\mathrm{mL}$ enant.1	Recovery % enant.1	Found $\mathrm{\mu g}/\mathrm{mL}$ enant.2	Recovery % enant.2
50.00	49.71	99.42	50.30	100.60	50.00	49.52	99.04	50.47	100.94
100.00	100.18	100.18	99.74	99.74	100.00	100.26	100.26	101.24	101.24
150.00	150.44	100.29	150.58	100.39	150.00	150.51	100.34	152.41	101.61
180.00	177.29	98.49	179.81	99.89	180.00	180.19	100.11	178.59	99.22
200.00	203.65	101.83	203.77	101.89	200.00	201.17	100.59	200.79	100.40
220.00	219.84	99.93	218.36	99.25	220.00	219.41	99.73	219.24	99.65
250.00	248.91	99.56	249.21	99.68	250.00	248.08	99.23	249.29	99.72
Mean		99.96		100.21			99.90		100.40
R.S.D		1.022		0.867			0.587		0.898

**Table 7:** Accuracy of the proposed HPLC method using mobile phase n-hexane: ethanol: 0.1% DEA (75: 25: 0.1 by volume) for quantitative determination of carvedilol and atenolol enantiomers

Carvedilol					Atenolol				
Taken $\mathrm{\mu g}/\mathrm{mL}$	Found $\mathrm{\mu g}/\mathrm{mL}$ enant.1	Recovery % enant.1	Found $\mathrm{\mu g}/\mathrm{mL}$ enant.2	Recovery % enant.2	Taken $\mathrm{\mu g}/\mathrm{mL}$	Found $\mathrm{\mu g}/\mathrm{mL}$ enant.1	Recovery % enant.1	Found $\mathrm{\mu g}/\mathrm{mL}$ enant.2	Recovery % enant.2
20.00	19.92	99.60	19.86	99.30	50.00	50.09	100.18	50.40	100.80
40.00	40.77	101.93	39.99	99.98	100.00	101.69	101.69	101.96	101.96
60.00	59.32	98.87	60.15	100.25	150.00	150.56	100.37	147.22	98.15
72.00	72.43	100.60	71.44	99.22	180.00	179.34	99.63	177.50	98.61
80.00	80.96	101.20	81.58	101.98	200.00	202.50	101.25	202.28	101.14
88.00	87.38	99.30	87.16	99.05	220.00	218.22	99.19	218.80	99.45
100.00	100.52	100.52	99.81	99.81	250.00	249.31	99.72	251.83	100.73
Mean		100.29		99.94			100.29		100.12
R.S.D		1.088		1.000			0.898		1.405

System suitability parameters of the proposed HPLC methods are shown in Tables 1 and 2. Robustness of the proposed method was investigated by analysis of samples under deliberate change in flow rate 1 ± 0.1 mL/min and temperature 25 °C ± 1 °C, the results are shown in Tables 3 and 4.

##### Precision

*Repeatability* For testing repeatability, the procedure under linearity was used for the analysis of three concentrations of each drug three times on the same day. %RSD value was calculated as shown in Tables 3 and 4.

*Intermediate precision* For testing intermediate precision, the same procedure was repeated on three successive days for assaying three freshly prepared solutions of each drug. %RSD values were computed as shown in Tables 3 and 4.

##### Limits of detection and quantification

Limit of detection (LOD) was calculated using the following equation

$$ {\text{LOD }} = { 3}.{3 } \times {\text{ SD}}/{\text{S}} $$

While Limit of quantification was calculated using the following equation

$$ {\text{LOQ }} = { 1}0 \, \times {\text{ SD}}/{\text{S}} $$

where, SD is the standard deviation of regression residuals and S is the slope of calibration curves as shown in Tables 3 and 4.

#### Molecular docking study of the five pairs of enantiomers on Lux Cellulose-2 column

The mechanism of separation of different enantiomers on the polysaccharide-based chiral stationary phases is mainly dependent on the chiral recognition upon formation of reversible types of interactions to form transient complexes between CSP and the enantiomer. This is achieved by inclusion of the enantiomer within the grooves present in the stationary phase polymer [29, 30].

As reported by Okamoto and Ikai [31, 32], the chiral recognition abilities of cellulose phenylcarbamate CSPs is mainly controlled by the nature and the position of the substituents on the phenyl groups. The cellulose phenylcarbamates bearing electron-donating substituents, such as alkyl groups, or electron-withdrawing substituents, such as halogens, exhibit higher chiral recognitions than the non-substituted one. This effect can be justified by the inductive influence of these substituents on the polarity of the carbamate group and thus on the interaction between CSP and the racemates. The electron-donating substituents increase the electron density at the carbonyl oxygen of the carbamate groups while the electron-withdrawing substituents increase the acidity of the NH proton of the carbamate groups. In polysaccharides-based CSPs, three key regions of the pendant groups strongly contribute to the retention and selectivity of the enantiomers, namely the electrophilic amidic hydrogen (N–H), the nucleophilic carbonyl oxygen (C=O) and the π–π electronic cloud [33].

In order to determine and understand the chiral recognition mechanism that is dependent on the stereochemistry of different enantiomers, computational modeling was used which can help us to predict the optimum conformation of the enantiomer and the selector to form a stable complex and accordingly have an insight into the specific enantioseparation.

**Fig. 4:**
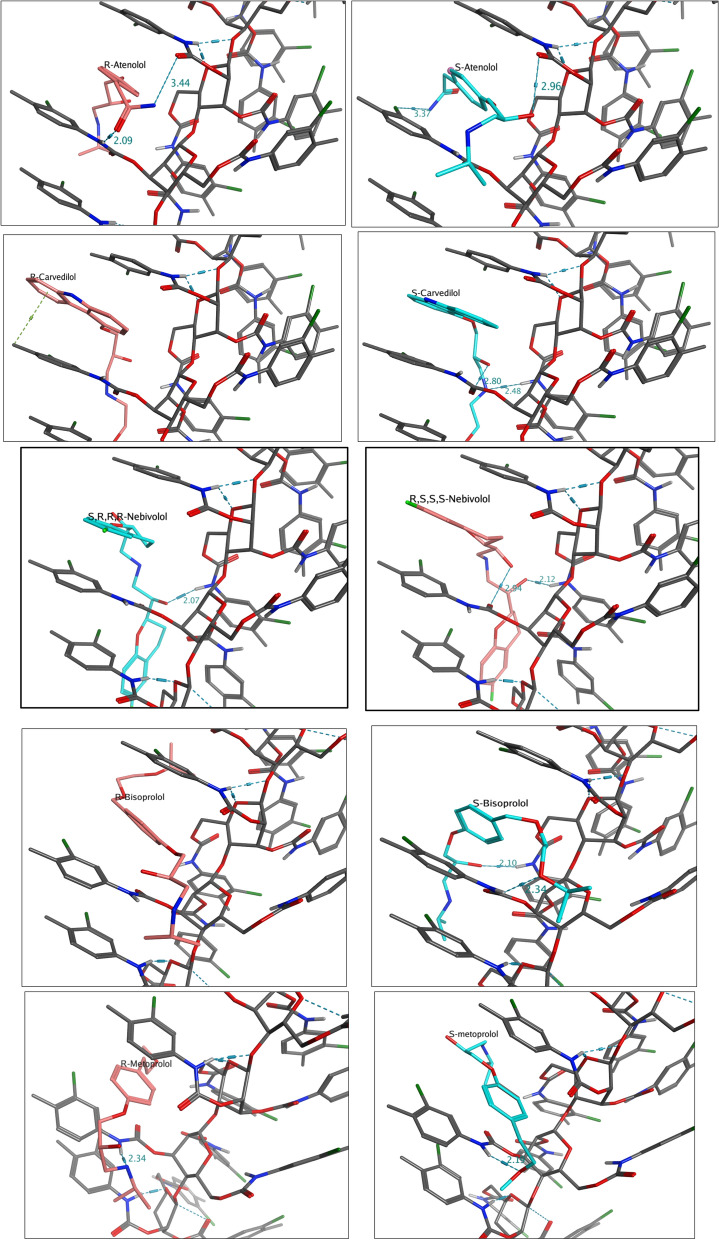
The 3D docking poses of the two enantiomers of the five studied analytes with the chiral stationary phase (CSP)

Molecular docking of the different enantiomers of the five analytes under investigation was carried out into the created grooves of CSP, Lux Cellulose-2, as a simulation of the binding process prior to chiral separation, using Molecular Operating Environment (MOE, 2020.0901) software. The docking results and the docking scores of the ten enantiomers with Lux Cellulose-2 are listed in Table 8. The 3D interaction of the enantiomers with the chiral stationary phase is shown in Fig. 4. More negative values reflect greater stability of the enantiomer-CSP binding as can be seen in Table 8, the docking scores of all the analytes are in negative sign, which means that the binding was enthalpically driven and spontaneous process. The binding energy (kcal/mol) for the ten enantiomers is in the following order: *S*-metoprolol (− 6.034) > *R*-metoprolol (− 6.105) > *R*-bisoprolol (− 6.24) > *S*-bisoprolol (− 6.36) > *S,R,R,R*-nebivolol (− 6.46) > *R,S,S,S*-nebivolol (− 6.72) > *R*-carvedilol (− 6.88) > *S*-carvedilol (− 7.22) > *S*-atenolol (− 8.367) > *R*-atenolol (− 8.667).

**Table 8:** Docking results of the enantiomers of five β‐adrenergic blockers with Lux-Cellulose-2 [Cellulose tris (3-chloro-4-methylphenylcarbamate)]

Compound	Docking score (Kcal/mol)	ΔΔE	Number of Interactions	Type of interactions	Binding site		Bond length (Å)
					Enantiomer	CSP	
*S*-metoprolol	− 6.034	0.071	1	H-bond	Methoxy oxygen	Carbamate NH	2.19
*R*-metoprolol	− 6.105		2	H-bond	NH nitrogen	Carbamate NH	2.34
				π-alkyl	Propyl side chain	3-Chloro-4-methylphenyl	4.13
*R*-bisoprolol	− 6.24	0.120	3	H-bond	Propoxy oxygen	Carbamate NH	2.34
				H-bond	Hydroxylic oxygen	Carbamate NH	2.10
				π-alkyl	Benzyl	3-Chloro-4-methylphenyl	2.90
*S*-bisoprolol	− 6.36		1	π-π- stacking	Benzyl	3-Chloro-4-methylphenyl	3.89
*S,R,R,R*-nebivolol	− 6.46	0.260	1	H-bond	Hydroxylic oxygen	Carbamate NH	2.07
*R,S,S,S*-nebivolol	− 6.72		2	H-bond	Hydroxylic oxygen	Carbamate NH	2.12
				H-bond	OH group	Carbamate C=O	2.94
*R*-carvedilol	− 6.88	0.340	1	π-alkyl	Carbazole	3-Chloro-4-methylphenyl	3.50
*S*-carvedilol	− 7.22		2	H-bond	NH of the side chain	Carbamate NH	2.11
				H-bond	OH group	Carbamate C=O	2.48
*S*-atenolol	− 8.367	0.300	2	H-bond	OH group	Carbamate C=O	2.96
				halogen bond	Amidic NH	Chloro substituent	3.37
*R*-atenolol	− 8.667		3	π-π- stacking	Phenyl group	3-Chloro-4-methylphenyl groups	3.83
				H-bond	C=O	Carbamate NH	2.23
				H-bond	Amidic NH	Carbamate C=O	2.12

By comparing the order of the above-mentioned docking scores to the elution trend of the analytes observed experimentally using both mobile phase systems, a good agreement can be found. In the first system using the ratio of hexane: ethanol was 60:40, by volume, the order of elution of the three analyzed pairs of enantiomers was metoprolol enantiomers (4.34 and 4.77 min) > carvedilol enantiomers (6.93 and 8.70 min) > atenolol enantiomers (13.70 and 20.30 min). This was consistent with the docking scores and it can be seen that atenolol enantiomers exhibited the strongest interaction with the CSP with the highest docking score and the longest retention on the stationary phase, while *S* (−)-metoprolol the weakest. While, in the second system when the ratio of hexane: ethanol was 75:25, by volume, the order of elution was metoprolol enantiomers (5.25 and 5.70 min) > nebivolol enantiomers (6.18 and 7.50 min) > carvedilol enantiomers (12.73 and 17.09 min) > atenolol enantiomers (30.82 and 47.55 min), showing an elution trend consistent with the order of the binding affinity which can be concluded from the obtained docking scores.

To further relate the binding energy with enantioselectivity, the difference in the binding energies of the pair of enantiomers ΔΔE (kcal/mol) was also evaluated. The absolute ΔΔE values (kcal/mol) were 0.071, 0.12, 0.26, 0.34 and 0.30 for metoprolol, bisoprolol, nebivolol, carvedilol and atenolol, respectively.

Further, chiral recognition mechanism can be understood from the 3D docking figures of enantiomers (Fig. 4 and Additional file 1: Figures S1-S10). The detailed information about each complex of the enantiomer with the selector is summarized in Table 8. The grooves that are present in the CSP contain carbonyl, amino and 3-chloro-4-methylphenyl residues, which can act as the main binding regions of the selector. These groups can interact with different groups of the ten enantiomers through H-bonding, π-π- -interactions, and hydrophobic interactions. As apparent, hydrogen bonding and hydrophobic interactions were mainly responsible for the enantioseparation.

The phenyl group of *R*-atenolol, the mostly retained enantiomer under the two experimental conditions, appeared sandwiched between the two 3-chloro-4-methylphenyl groups of CSP through π-π-stacking, in addition to two hydrogen bonds: the first one formed between the carbonyl of atenolol and the hydrogen of the amino group of CSP and the other one was attaching the hydrogen of the amidic nitrogen and the carbonyl of the CSP carbamate group. While *S*-atenolol was deeply buried in the CSP cavity between the two 3-chloro-4-methylphenyl groups and interacted with the selector through hydrogen bond between the hydrogen of the OH group of atenolol and the carbonyl group of CSP carbamate group in addition to a halogen bond between the amidic NH of atenolol and the chloro-substituent of the selector.

By comparing the two enantiomers of carvedilol, the carbazole ring of both appeared buried in the CSP cavity but the *S*- enantiomer appeared to be more retained on the stationary phase as it interacted through two additional hydrogen bonding: NH of the side chain of carvedilol with the amino group of CSP carbamate and the OH group of carvedilol with another CSP carbonyl group.

*S,R,R,R*- and *R,S,S,S*-nebivolol, both enantiomers exerted the same hydrogen bonding interactions between the hydroxylic oxygen of nebivolol and the NH of the CSP carbamate group. In both enantiomers, one of the benzopyran rings was sandwiched between two 3-chloro-4-methylphenyl groups of the chiral stationary phase. *R,S,S,S*-Nebivolol showed an additional H-bond between its other OH group and the CSP carbonyl group, which may contribute to the difference in retention time between the two enantiomers.

In case of bisoprolol, the chiral separation might be derived from the difference in interactions as the *S*-enantiomer was docked in the CSP groove via two hydrogen bonding interactions, between the oxygen atom of the propoxy group and the OH group of bisoprolol as H-acceptors and the NH of two CSP carbamates as H-donors. Both enantiomers were trapped in the groove through π-alkyl stacking of bisoprolol benzyl group and the two 3-chloro-4-methylphenyl groups of the stationary phase.

On the other hand, *S*-metoprolol which was the least retained had only one point of interaction with the CSP, via hydrogen bonding between the oxygen of the methoxy group of metoprolol and the NH of the CSP carbamate group. However, *R*-metoprolol had greater retention due to one hydrogen bond between the nitrogen of the amino group of the analyte and the NH of the CSP carbamate group and a hydrophobic interaction involving π-alkyl-bonding of 3-chloro-4-methylphenyl with the propyl side chain.

### Molecular dynamics of the five pairs of enantiomers on Lux Cellulose-2 column

A computer simulation, such as molecular mechanics (MM) and molecular dynamics (MD) calculations, is a useful and effective approach for the qualitative understanding of the chiral recognition on the polysaccharide-based CSPs, for the prediction of the chromatographic behavior.

In the molecular dynamic study, all the trajectory conformations were compared from 0 to 600 ps to evaluate the stability of the binding and the effect of solvation on the interaction behavior over the time of the inclusion complex of CSP with the five pairs of enantiomers. Cellulose carbamate-based polymers are mainly characterized by two main structural features: the polysaccharide backbone, where conformational chirality depends on the helical twist generated by the glycosidic linkage that forms the polymeric chain and the hanging groups which allow for the expansion of the polymer and accordingly the formation of the chiral groove. This groove consists of a polar layer containing the carbamate groups and able to exert polar interactions, and a hydrophobic layer containing substituted methyl and chloro- aromatic rings located outside the polymer groove and able to exert π-π- and hydrophobic interactions [34, 35]. Thus, it can be inferred that the flexibility of CSP played a dominative role in the chiral recognition (For more information, see Additional file 1: Figures S11-S40).

For R-atenolol, at 0 ps, the enantiomer was only interacted with one ethanol molecule through H-π interaction. While at 100 ps, S-atenolol interacted with CSP through hydrogen bonding with the carbonyl group of CSP in addition to H-π interaction with one of the 3-chloro-4-methylphenyl groups, keeping HB interactions with the solvent molecules. Another enantiomer-CSP interaction appeared at 600 ps through hydrogen bonding between the CO of S-atenolol and the NH group of CSP as hydrogen donor, which may attribute to the retention of the S-enantiomer of atenolol at CSP (Additional file 1: Figures S11-S13).

For S-atenolol, at 0 ps, it was deeply buried into the CSP cavity between the two 3-chloro-4-methylphenyl groups, and it interacted with the CSP through hydrogen bond between the hydrogen of the OH group of atenolol and the carbonyl group of CSP carbamate group in addition to a hydrogen bond between the amidic NH of atenolol and the oxygen of an ethanol molecule. In addition, there was a H-π interactions between S-atenolol phenyl ring and one 3-chloro-4-methylphenyl group of CSP. At 100 ps, S-atenolol molecule began to interact more with the surrounding ethanol molecules keeping only the HB interaction with CSP while at 600 ps, S-atenolol returned back to CSP cavity interacting through H-π interaction only, (Additional file 1: Figures S14-S16).

For carvedilol enantiomers, the carbazole ring of the R-enantiomer was buried in the CSP cavity showing nearly the same interactions with CSP through the poses from 0 to 600 ps (One HB and two H-π interactions) (Figures S17-S19). On the other hand, The S-enantiomer performed nearly no interaction with CSP and much higher number of interactions with the solvent molecules (Additional file 1: Figures S20-S22).

The *S,R,R,R*-nebivolol exerted less interactions with CSP than in the molecular docking study as it only interacted through H-π interaction in all the trajectory conformations and no HB interactions were identified while it exhibited many HB interactions with the solvent molecules revealing the significant effect of the solvent on the formation of enantiomer-CSP complex (Additional file 1: Figures S23-S25). On the contrary, the *R,S,S,S*-nebivolol performed a hydrogen bonding interaction between the hydroxylic oxygen of nebivolol and the CO of the CSP carbamate group, in addition to three H-π interactions, but all these interactions decreased with time, (Additional file 1: Figures S26-S28).

In case of bisoprolol, both enantiomers exhibited nearly the same pose as via docking study without any HB interactions with CSP, (Additional file 1: Figures S29-S34).

The differential affinity of metoprolol enantiomers was also persistent in dynamic study as *R*-metoprolol exhibited hydrogen bond interactions that was stable with time, (Additional file 1: Figures S35-S37) while *S*-metoprolol didn’t undergo any HB interactions with CSP while interacted with the solvent molecules, (Additional file 1: Figures S38-S40).

## Conclusion

In this work, enantioseparation and quantification of five β-adrenergic blockers on a Lux-Cellulose-2 (cellulose tris (3-chloro-4-methyl phenyl carbamate) column was investigated. The quantification is important for future analysis of pharmaceutical products, lab mixtures and in quality control laboratories. The first mobile phase used for separation and quantification of three β-adrenergic blockers, namely, bisoprolol, carvedilol and atenolol. By changing hexane: ethanol ratio of the first mobile phase, separation and quantification of four β-adrenergic blockers, namely, metoprolol, nebivolol, carvedilol and atenolol were achieved. It could be predicted that Lux-Cellulose-2 column could be useful for the enantioseparation and quantification of the drugs in quality control laboratories. Simulation studies of the drugs on chiral selector were done to study the chiral recognition mechanism of the enantiomers. The molecular docking and dynamics results were in accordance with the chromatographic separation results. So, the reported modeling approach could be used to explain the chiral recognition mechanisms of racemic drugs by HPLC on Lux-Cellulose-2 (cellulose tris (3-chloro-4-methyl phenyl carbamate).

## Supplementary Information

**Additional file 1.**: Additional Figures

## Data Availability

All data generated or analyzed during this study are included in this published article and its additional information files.

## Abbreviations

CSPs: Chiral stationary phases
MD: Molecular dynamics
MOE: Molecular operating environment
