# Supplementary material for: Enantioseparation, quantification, molecular docking and molecular dynamics study of five β-adrenergic blockers on Lux-Cellulose-2 column
Source: BMC Chem. 2023 Mar 16;17(1):22. doi: 10.1186/s13065-023-00925-2 (PMC10018884; doi:10.1186/s13065-023-00925-2)
Supplement: Supplementary file 1 — Additional file 1.: Additional Figures [file 13065_2023_925_MOESM1_ESM.docx]

**Supplementary Data**

**Enantioseparation, quantification, molecular docking and dynamic study of five beta-adrenergic blockers on Lux-Cellulose-2 column**

**Ola Ahmed Saleh*^a^, Amr Mohamed Badawey ^b^, Hassan Y. Aboul-Enein*^a^, Marwa Ahmed Fouad ^c, d^**

^a^ Medicinal and Pharmaceutical Chemistry Department, Pharmaceutical and Drug Industries Research Institute, National Research Centre (ID: 60014618), P.O. 12622, Giza, Egypt, ^b^Analytical Chemistry Department, Faculty of Pharmacy, Cairo University, Kasr El-Aini St., Cairo 11562, Egypt, ^c^Pharmaceutical Chemistry Department, Faculty of Pharmacy, Cairo University, Kasr El-Aini St., Cairo 11562, Egypt, ^d^Pharmaceutical Chemistry Department, School of Pharmacy, NewGiza University, NewGiza, km 22 Cairo-Alexandria Desert Road, Cairo, Egypt.

**Figures showing 3D interaction of the five pairs of enantiomers on Lux Cellulose-2 column using Molecular dynamic simulation**


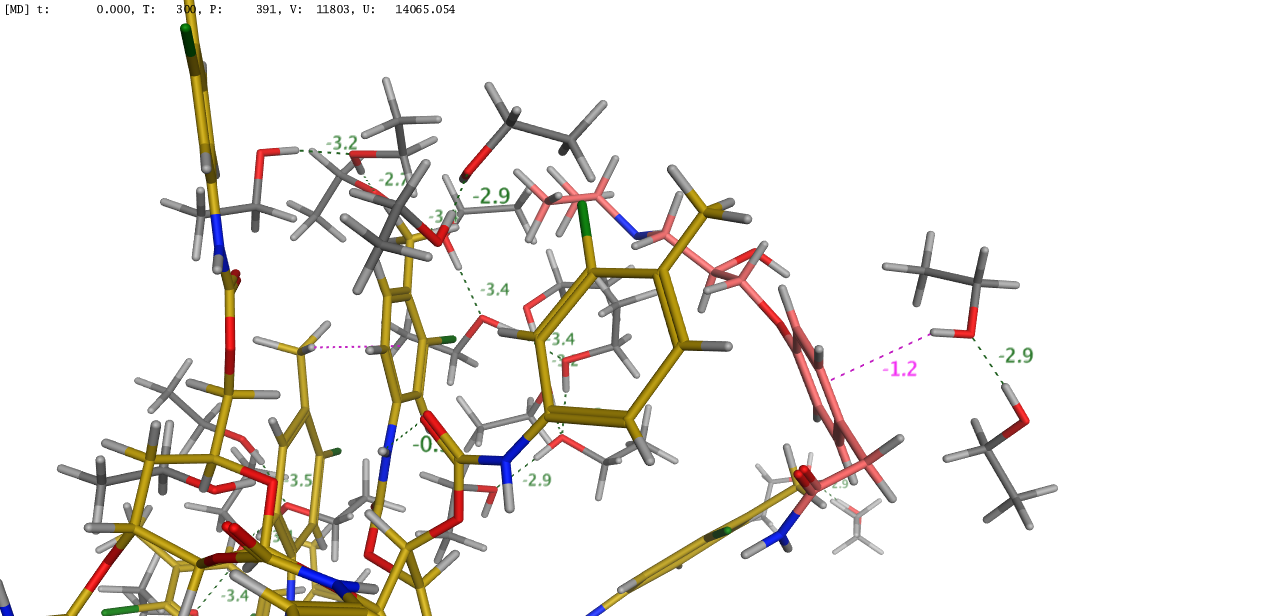


**Fig. S1:** Binding interactions of R-atenolol with CSP throughout dynamic simulation at 0 ps.


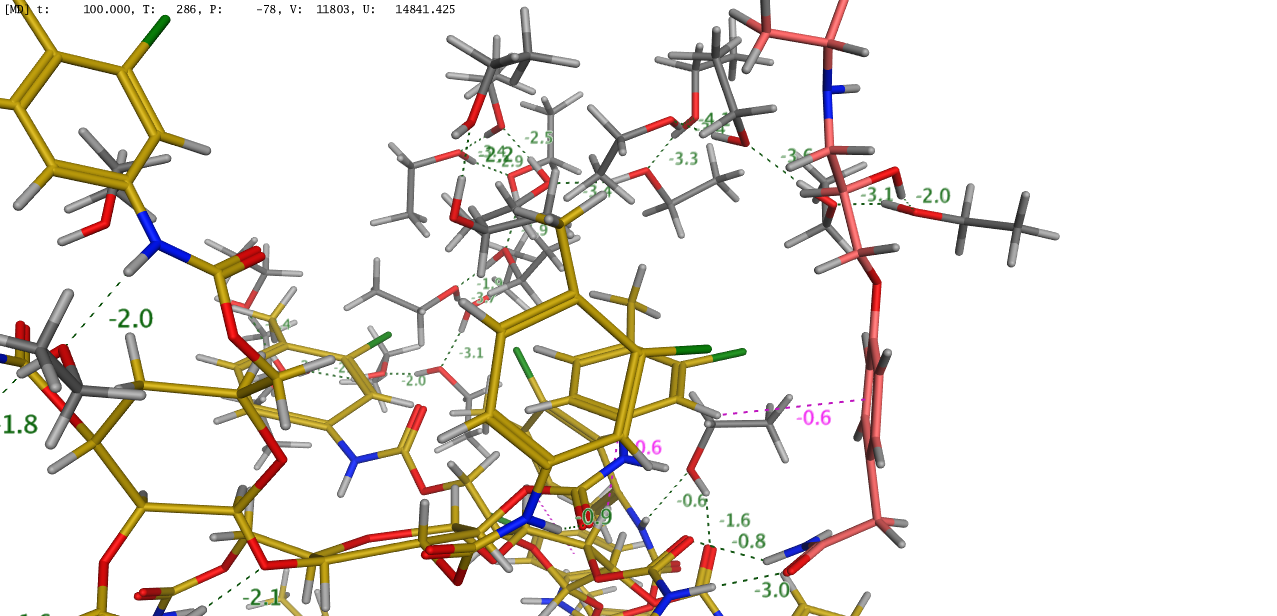


**Fig. S2:** Binding interactions of R-atenolol with CSP throughout dynamic simulation at 100 ps.


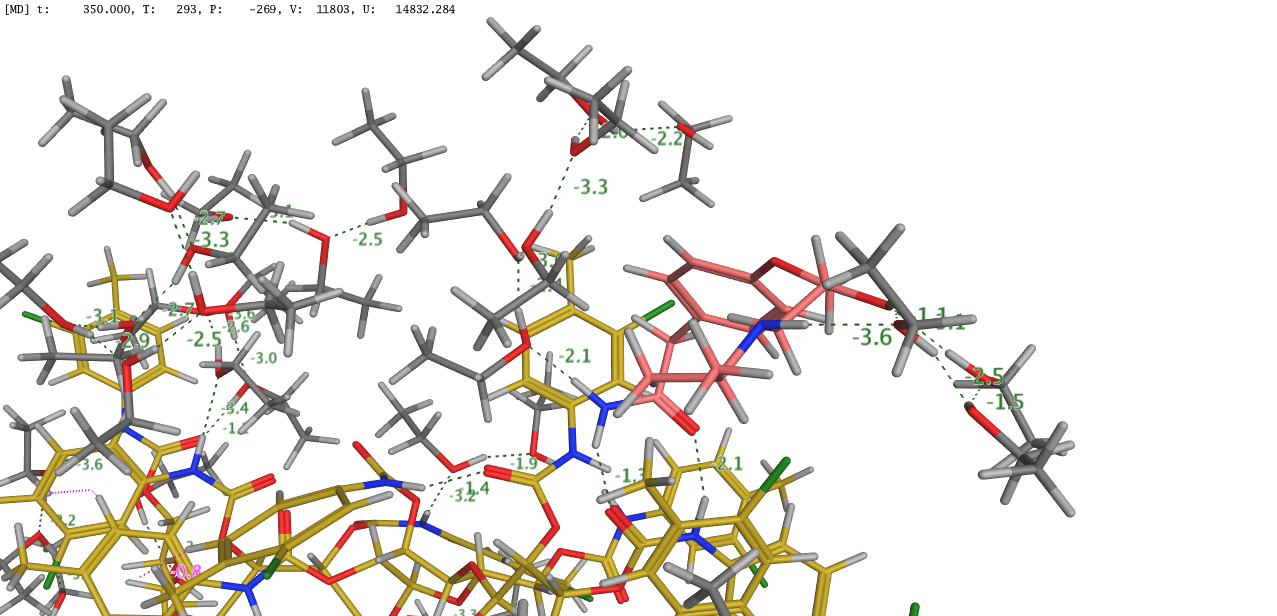


**Fig. S3:** Binding interactions of R-atenolol with CSP throughout dynamic simulation at 600 ps.


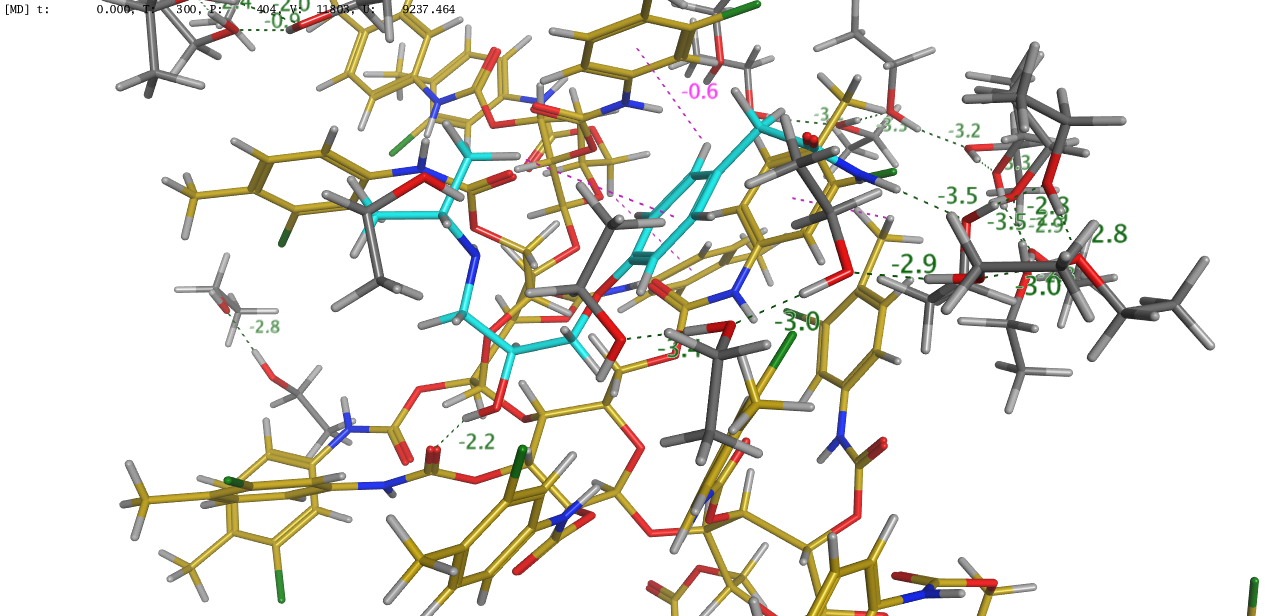


**Fig. S4:** Binding interactions of S-atenolol with CSP throughout dynamic simulation at 0 ps.


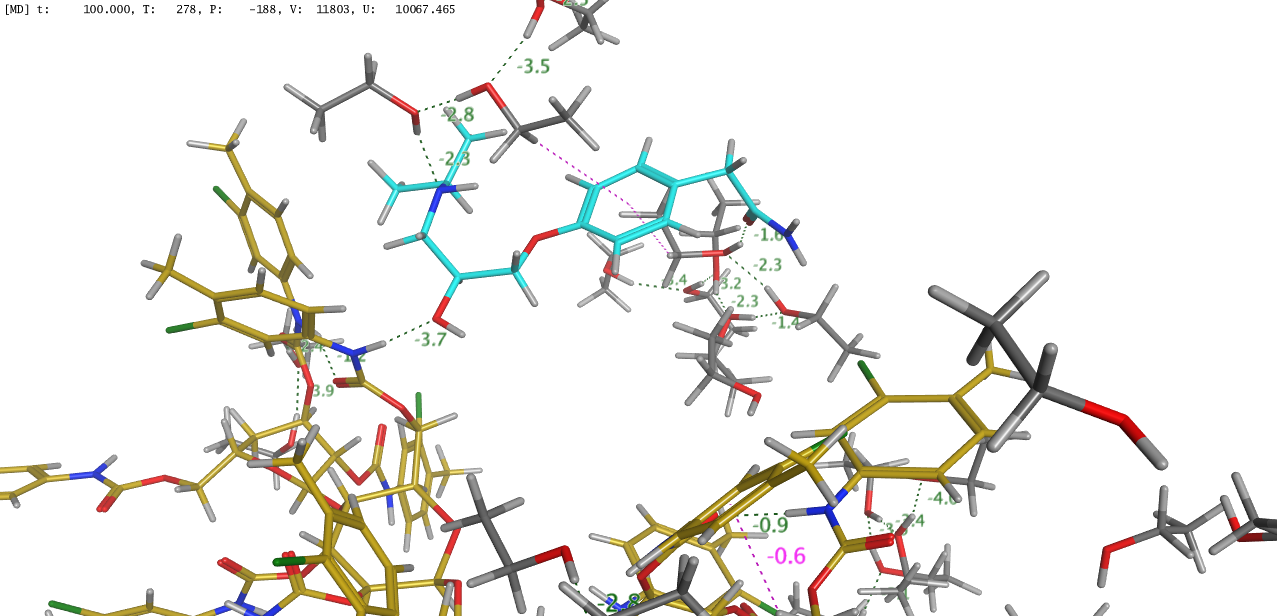


**Fig. S5:** Binding interactions of S-atenolol with CSP throughout dynamic simulation at 100 ps.


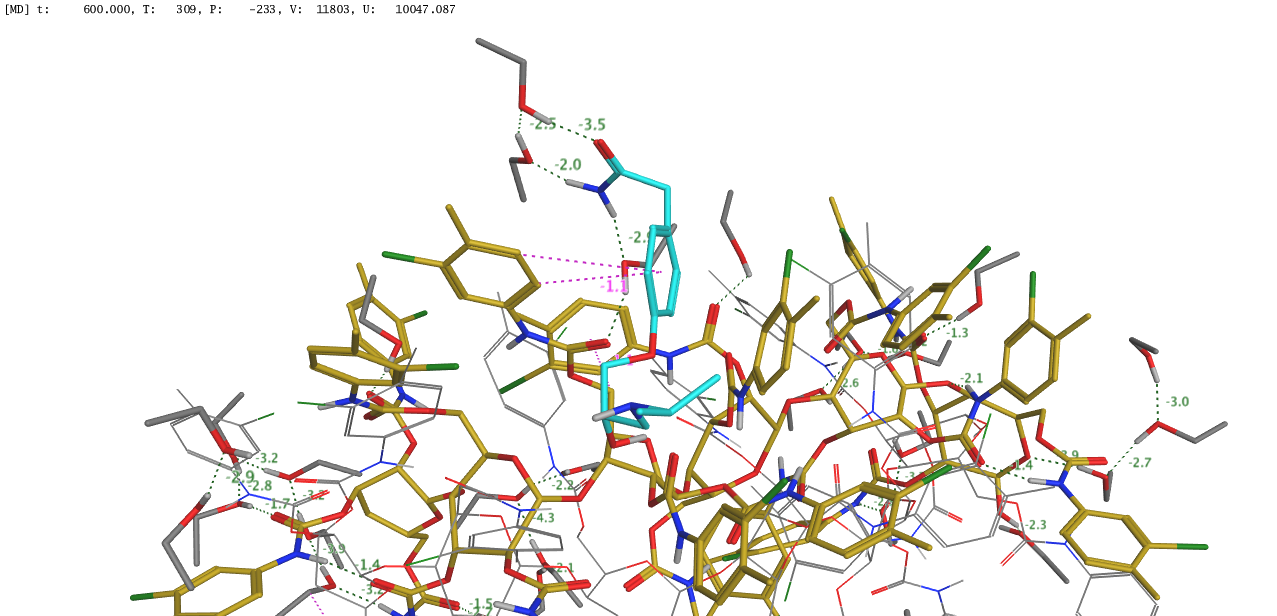


**Fig. S6:** Binding interactions of S-atenolol with CSP throughout dynamic simulation at 600 ps.


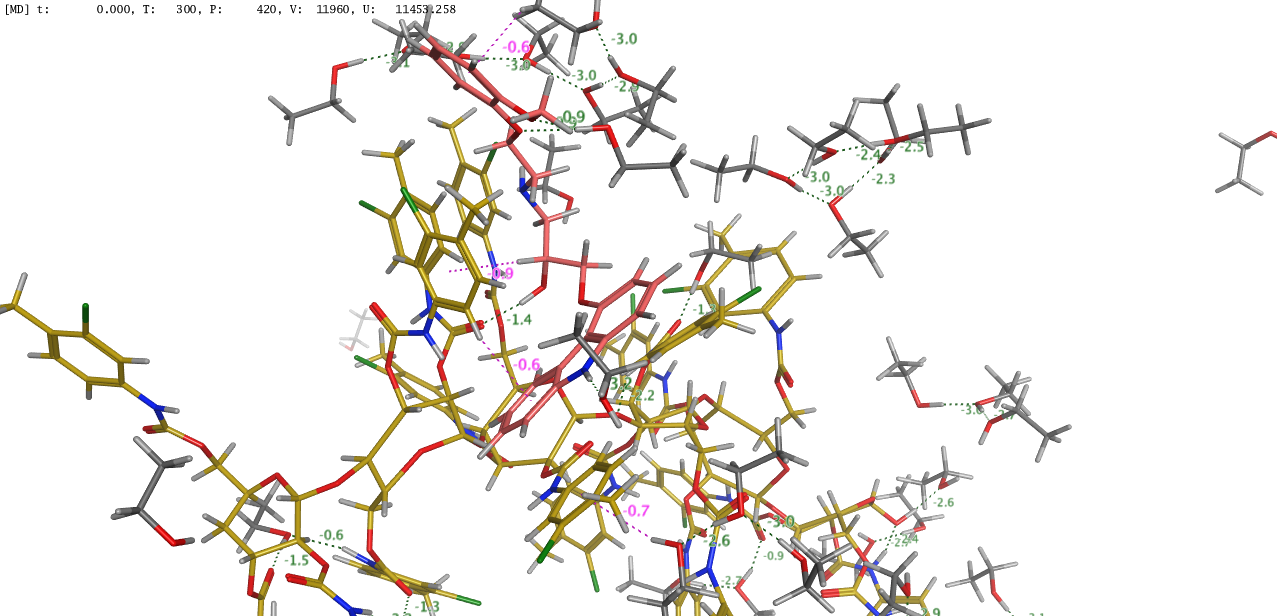


**Fig. S7:** Binding interactions of R-carvedilol with CSP throughout dynamic simulation at 0 ps.


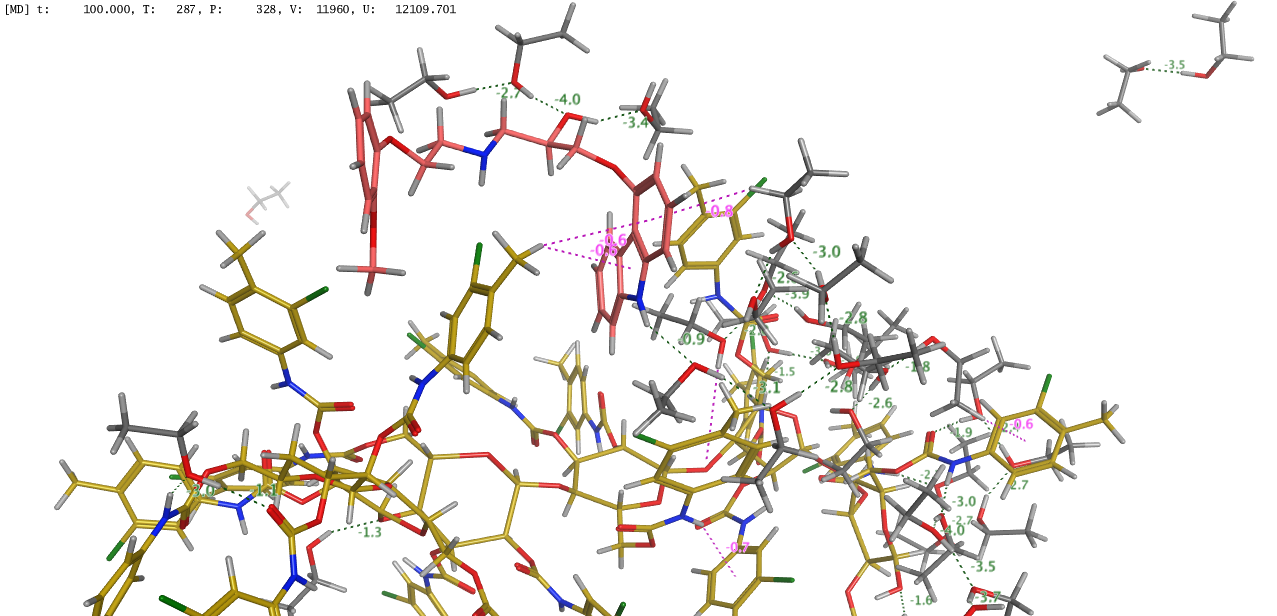


**Fig. S8:** Binding interactions of R-carvedilol with CSP throughout dynamic simulation at 100 ps.


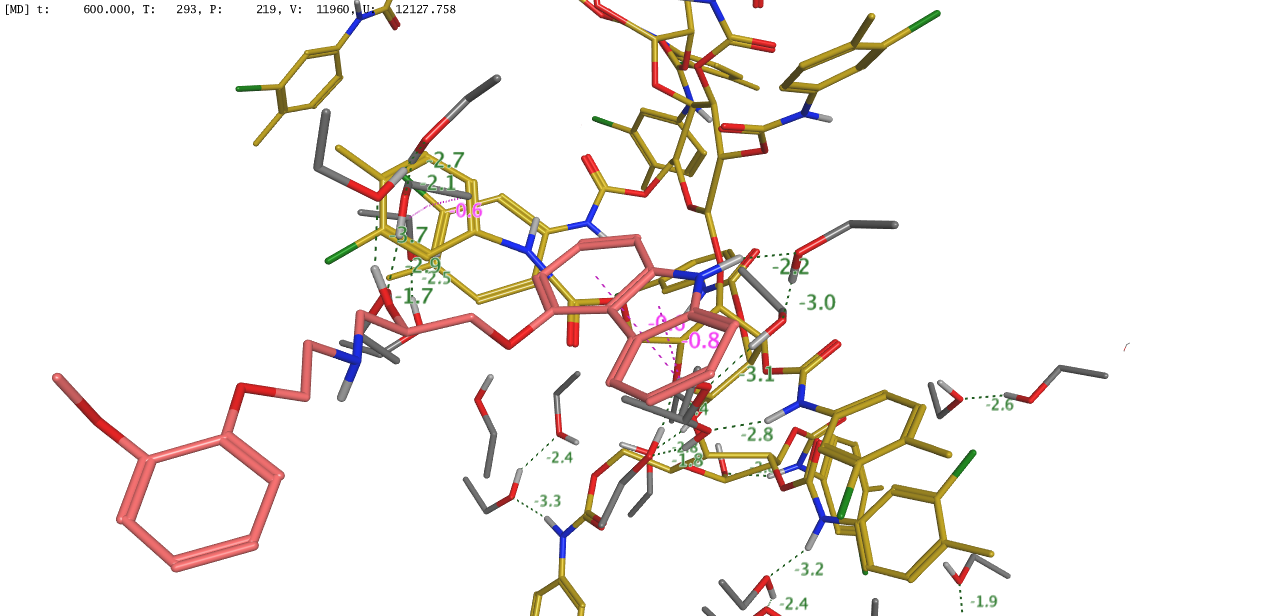


**Fig. S9:** Binding interactions of R-carvedilol with CSP throughout dynamic simulation at 600 ps.


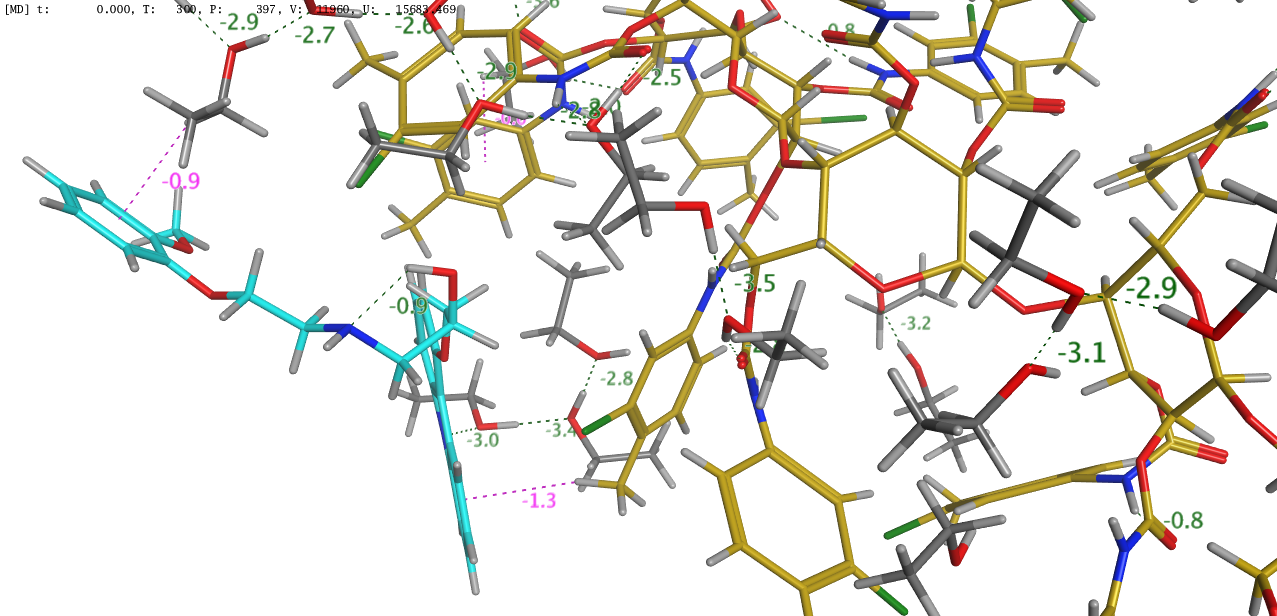


**Fig. S10:** Binding interactions of S-carvedilol with CSP throughout dynamic simulation at 0 ps.


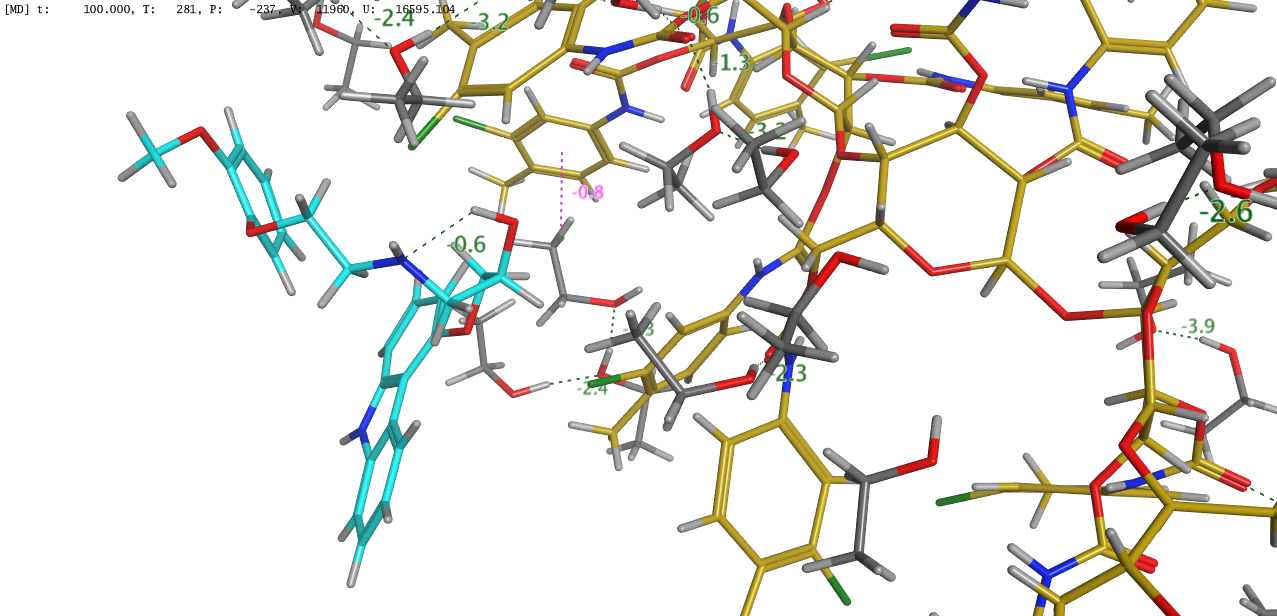


**Fig. S11:** Binding interactions of S-carvedilol with CSP throughout dynamic simulation at 100 ps.


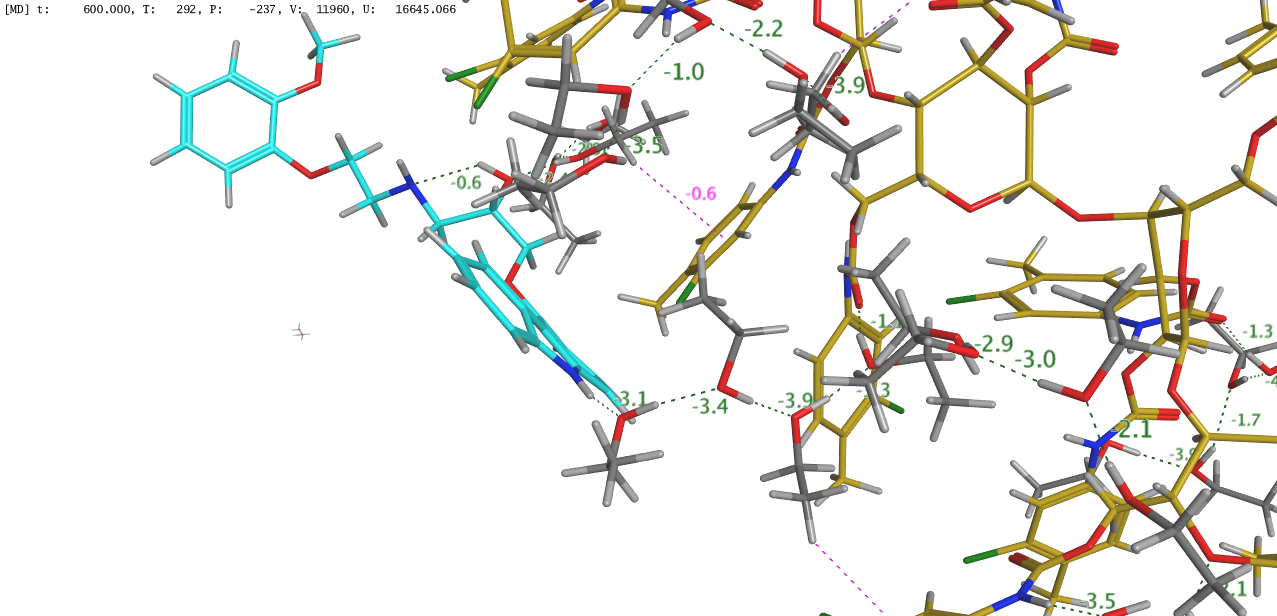


**Fig. S12:** Binding interactions of S-carvedilol with CSP throughout dynamic simulation at 600 ps.


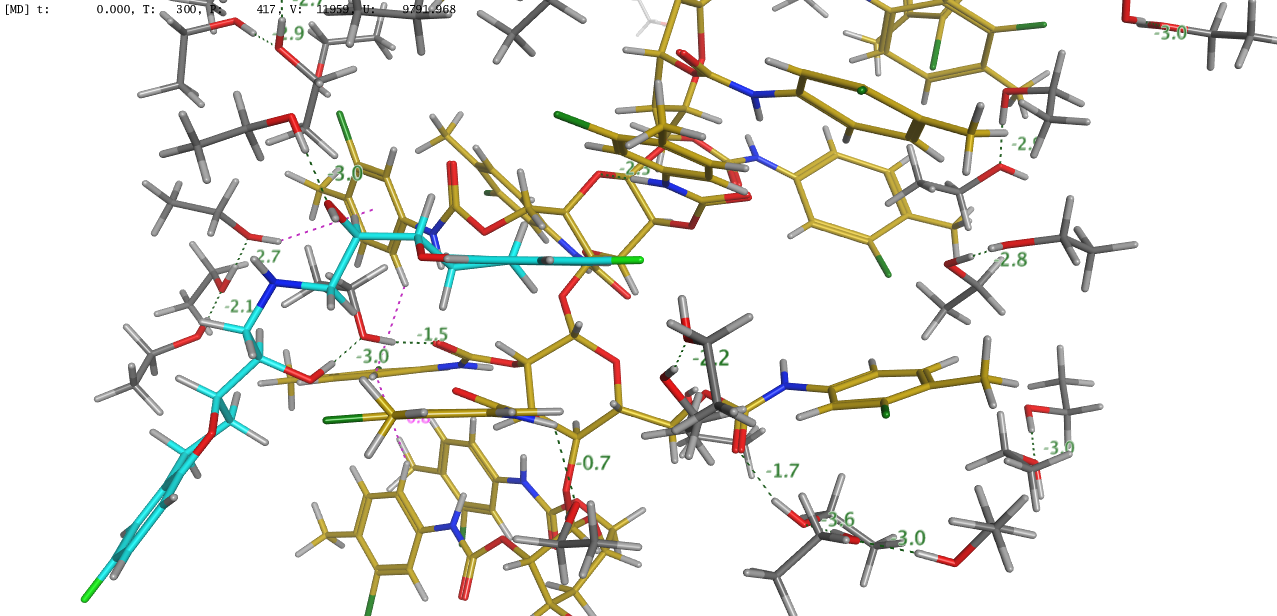


**Fig. S13:** Binding interactions of d-nebivolol with CSP throughout dynamic simulation at 0 ps.


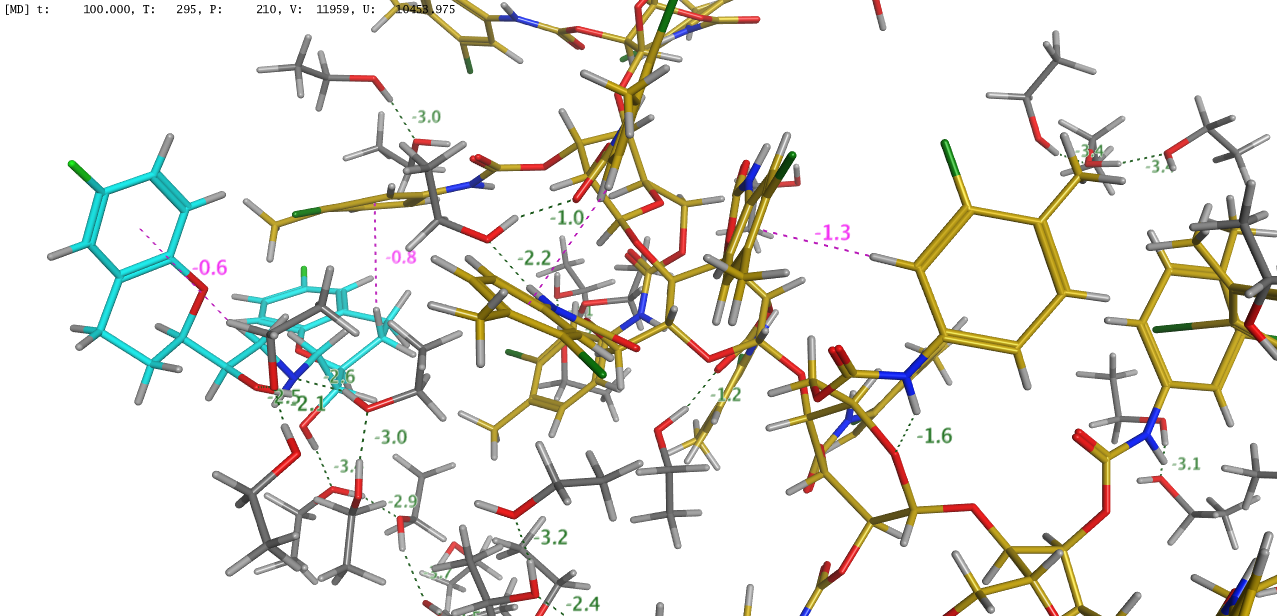


**Fig. S14:** Binding interactions of d-nebivolol with CSP throughout dynamic simulation at 100 ps.


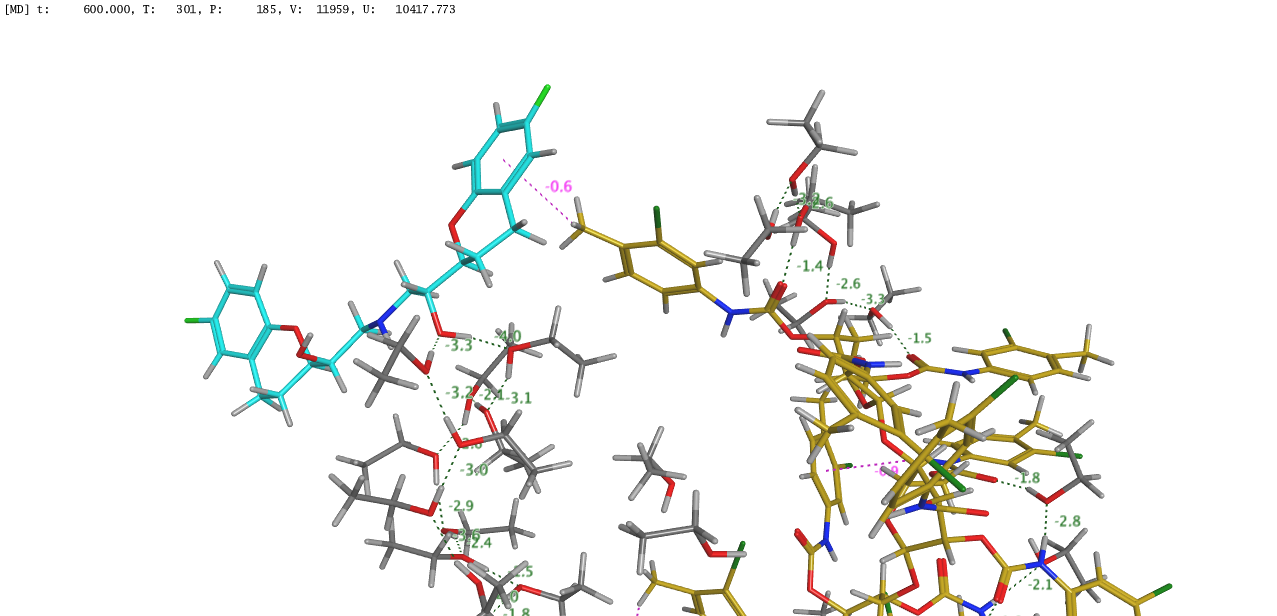


**Fig. S15:** Binding interactions of d-nebivolol with CSP throughout dynamic simulation at 600 ps.


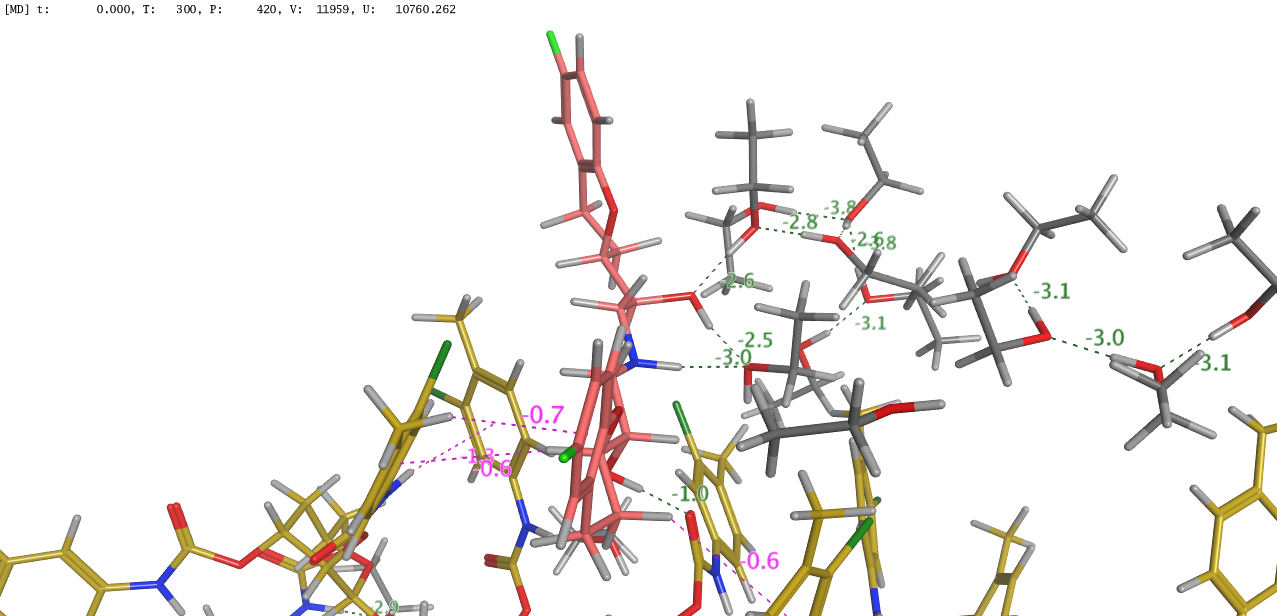


**Fig. S16:** Binding interactions of l-nebivolol with CSP throughout dynamic simulation at 0 ps.


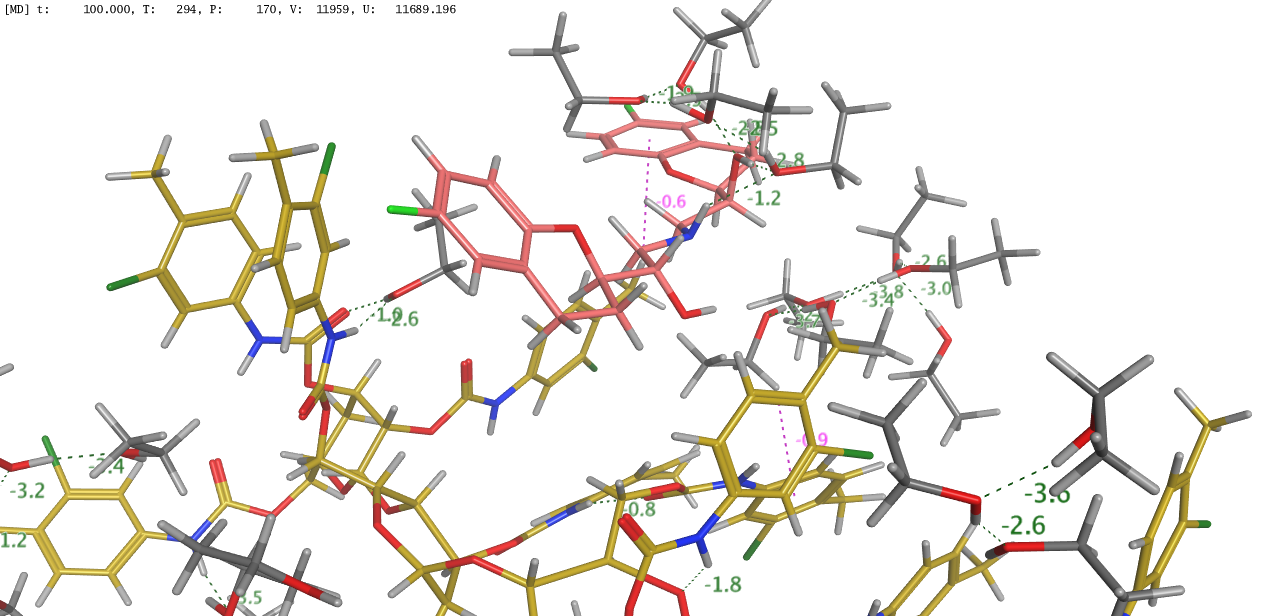


**Fig. S17:** Binding interactions of l-nebivolol with CSP throughout dynamic simulation at 100 ps.


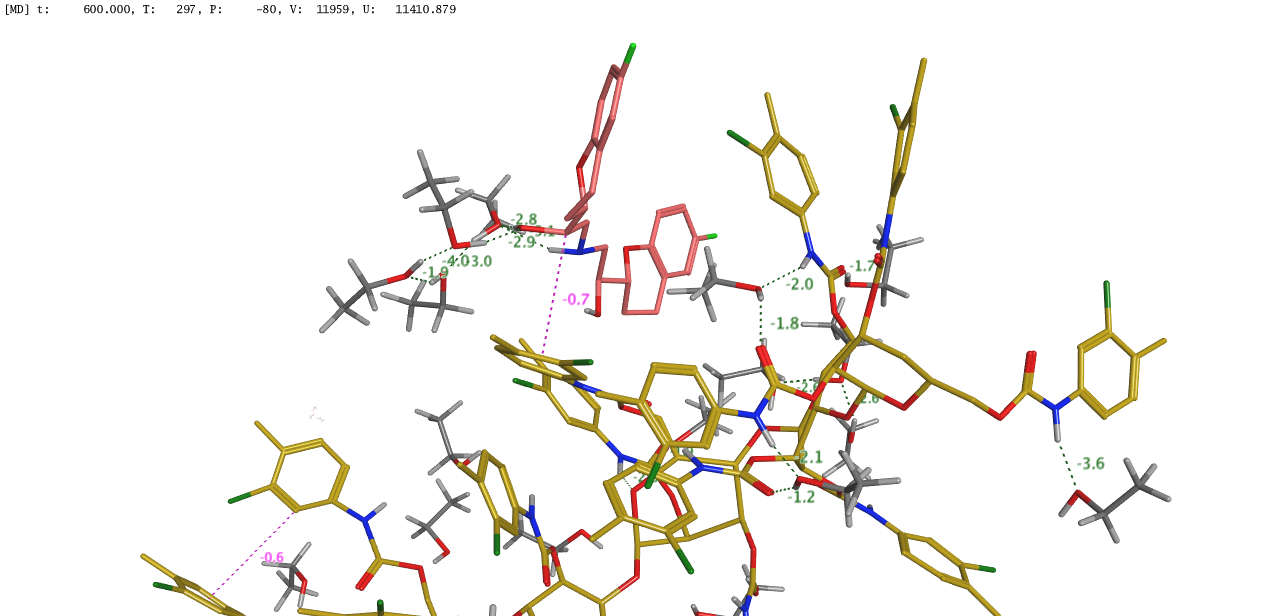


**Fig. S18:** Binding interactions of l-nebivolol with CSP throughout dynamic simulation at 600 ps.


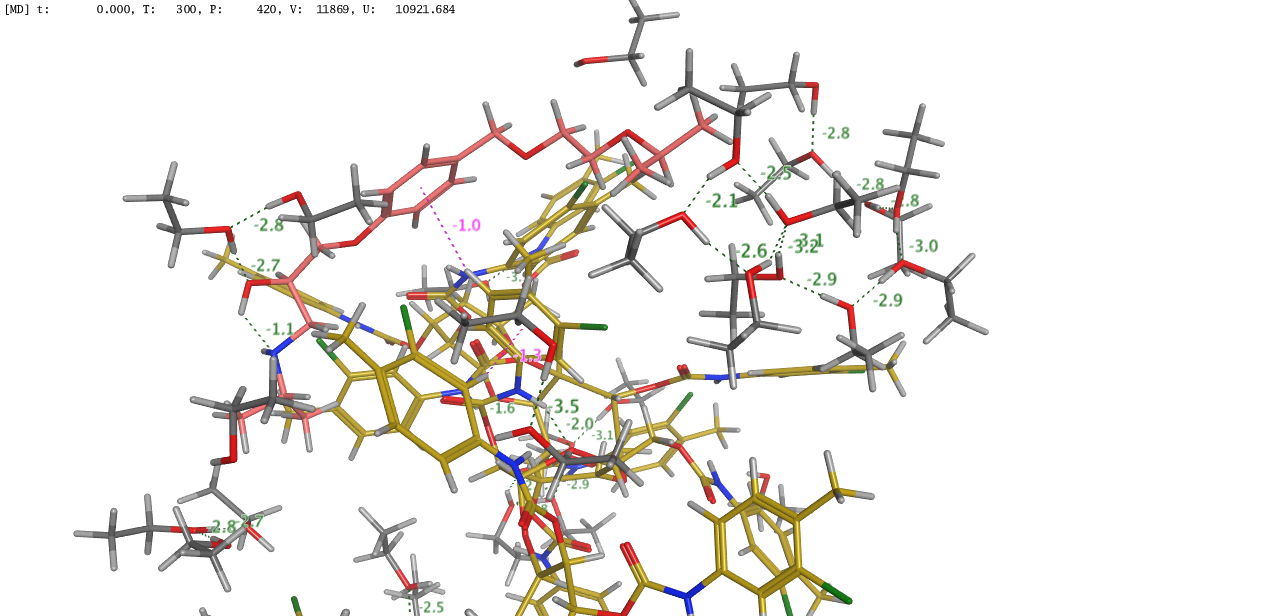


**Fig. S19:** Binding interactions of R-bisoprolol with CSP throughout dynamic simulation at 0 ps.


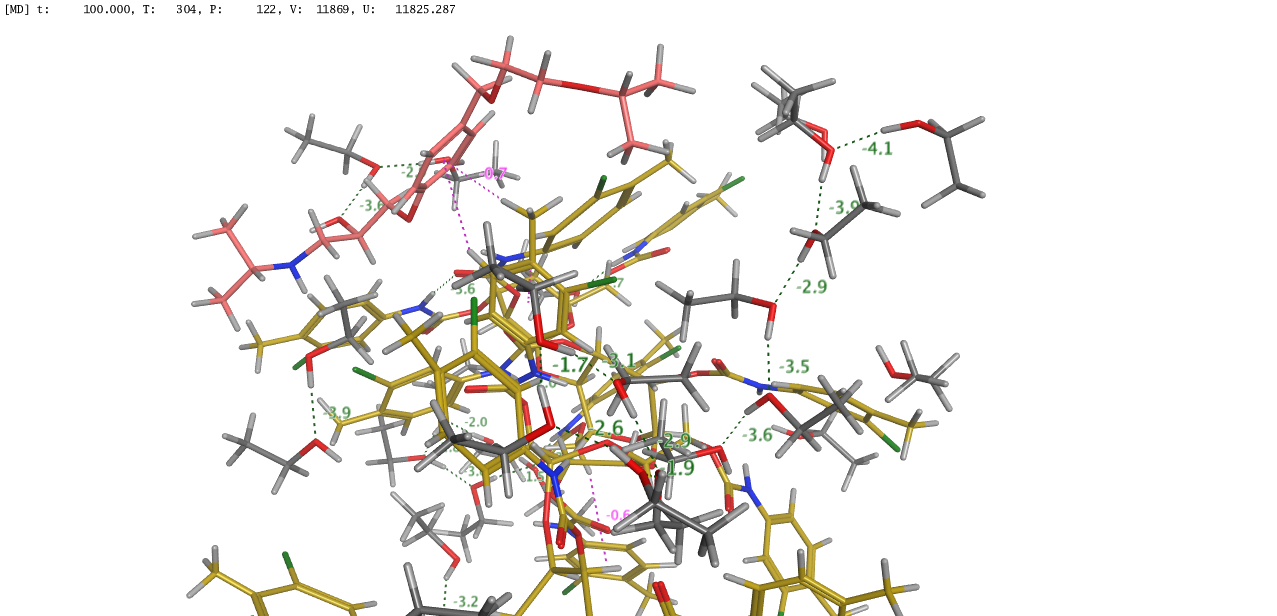


**Fig. S20:** Binding interactions of R-bisoprolol with CSP throughout dynamic simulation at 100 ps.


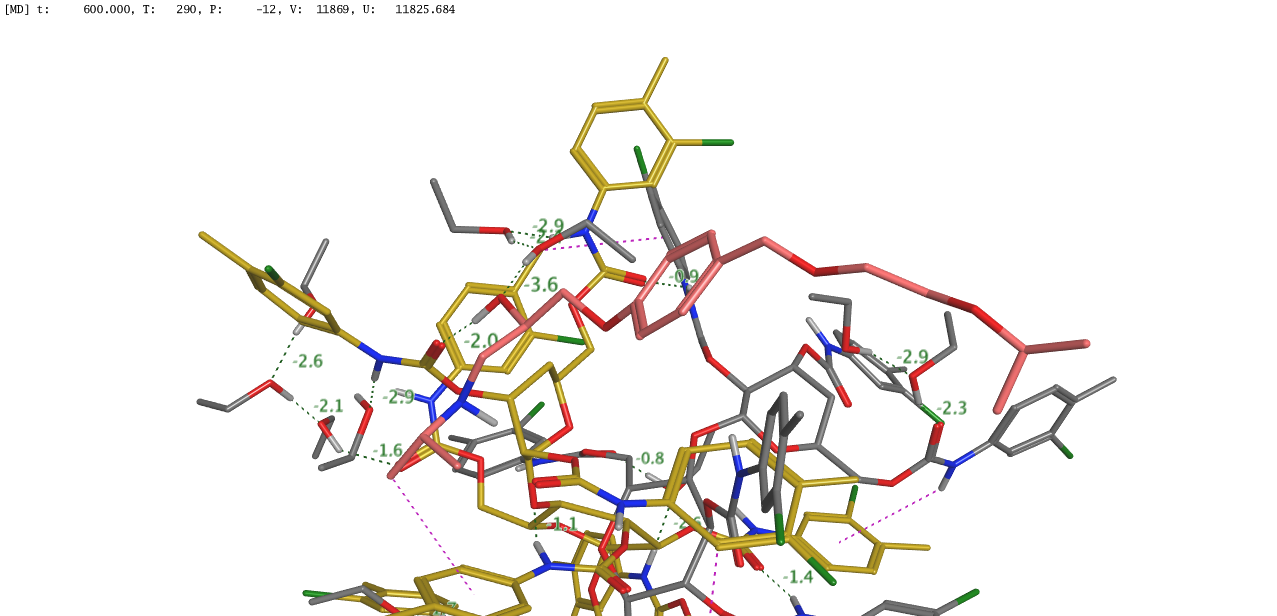


**Fig. S21:** Binding interactions of R-bisoprolol with CSP throughout dynamic simulation at 600 ps.


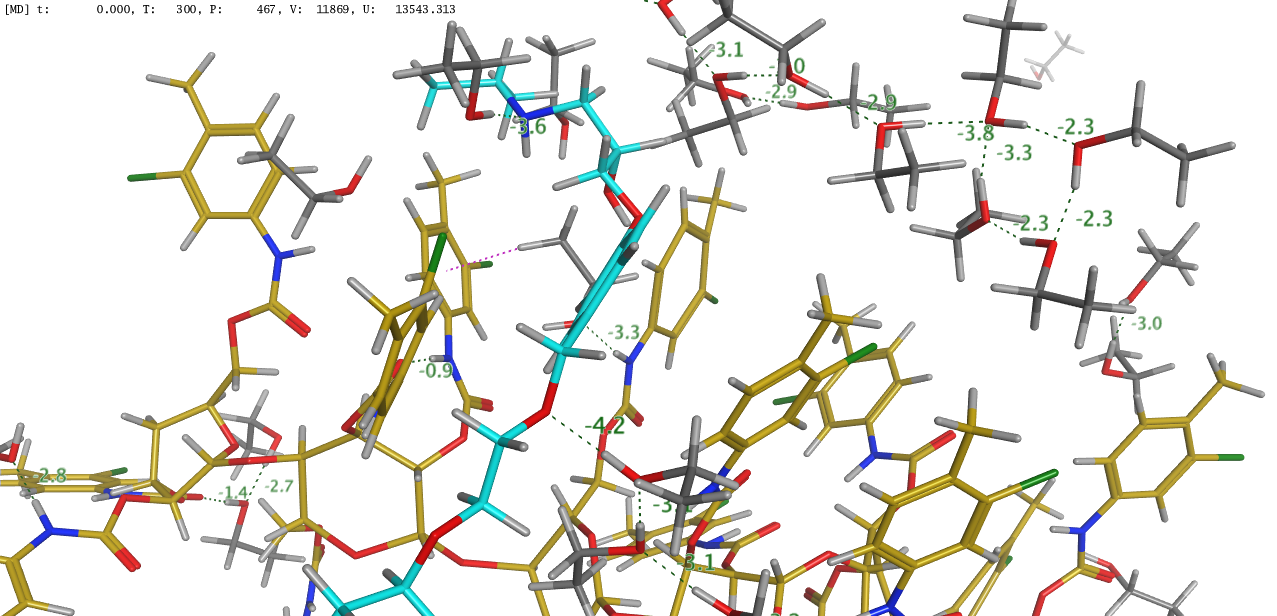


**Fig. S22:** Binding interactions of S-bisoprolol with CSP throughout dynamic simulation at 0 ps.


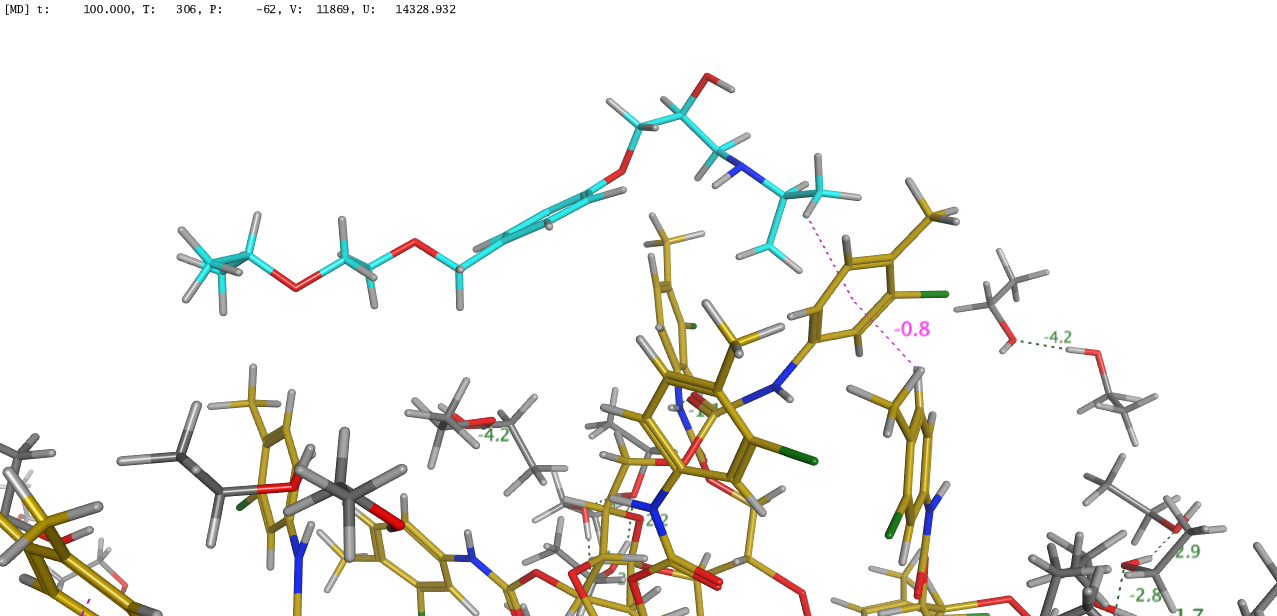


**Fig. S23:** Binding interactions of S-bisoprolol with CSP throughout dynamic simulation at 100 ps.


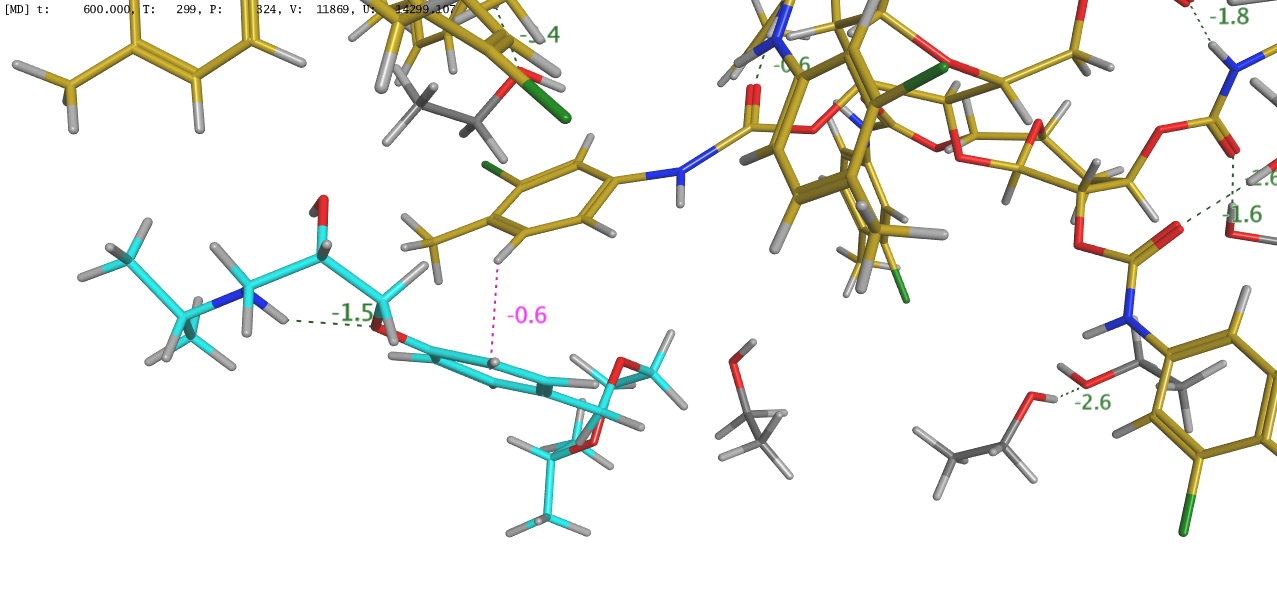


**Fig. S24:** Binding interactions of S-bisoprolol with CSP throughout dynamic simulation at 600 ps.


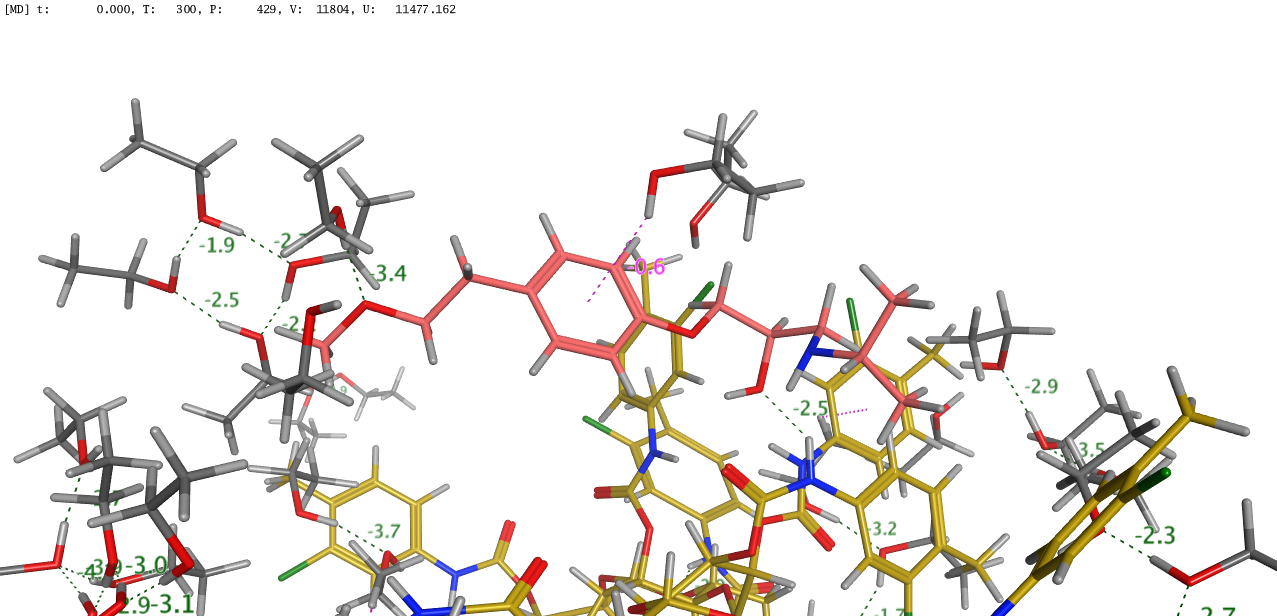


**Fig. S25:** Binding interactions of R-metoprolol with CSP throughout dynamic simulation at 0 ps.


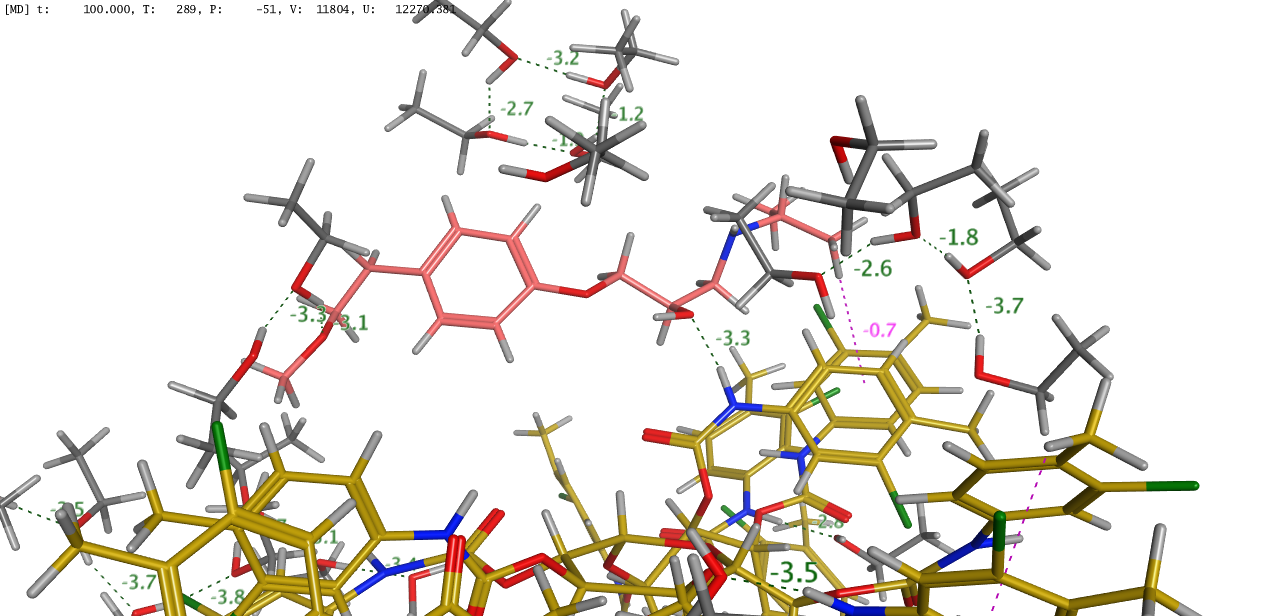


**Fig. S26:** Binding interactions of R-metoprolol with CSP throughout dynamic simulation at 100 ps.


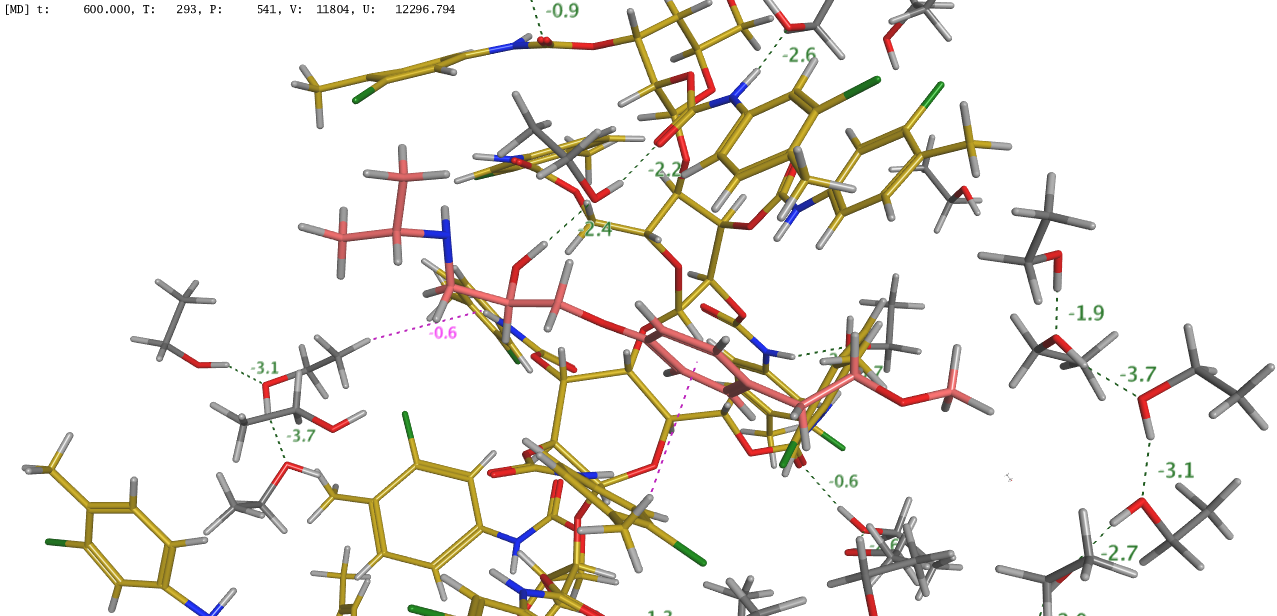


**Fig. S27:** Binding interactions of R-metoprolol with CSP throughout dynamic simulation at 600 ps.


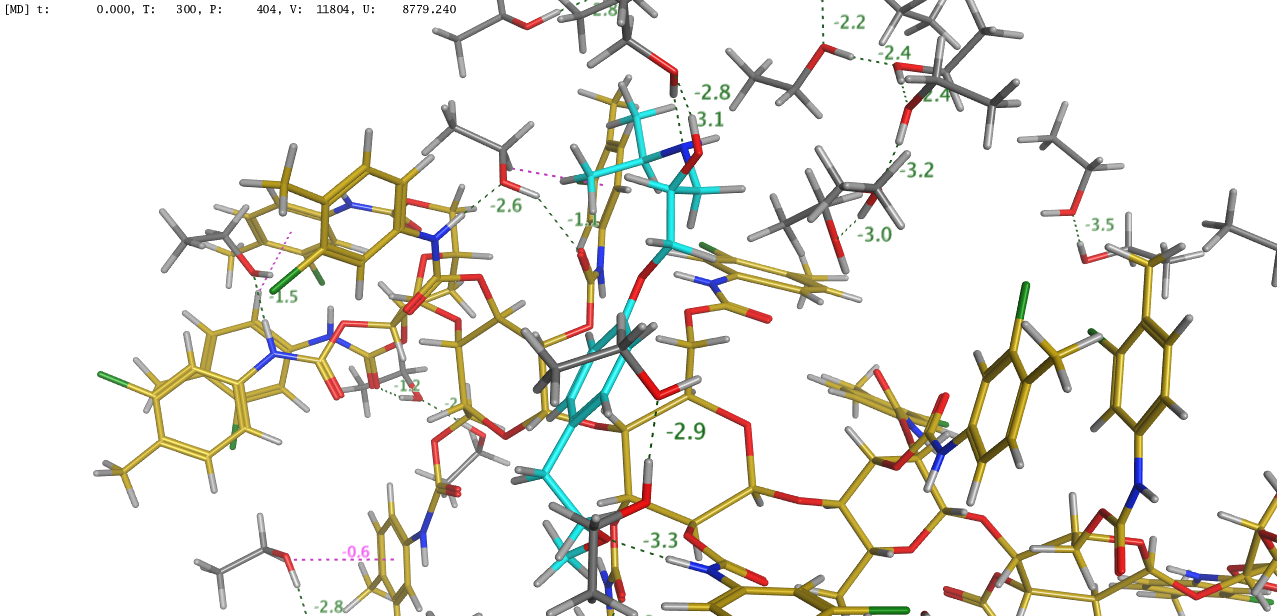


**Fig. S28:** Binding interactions of S-metoprolol with CSP throughout dynamic simulation at 0 ps.


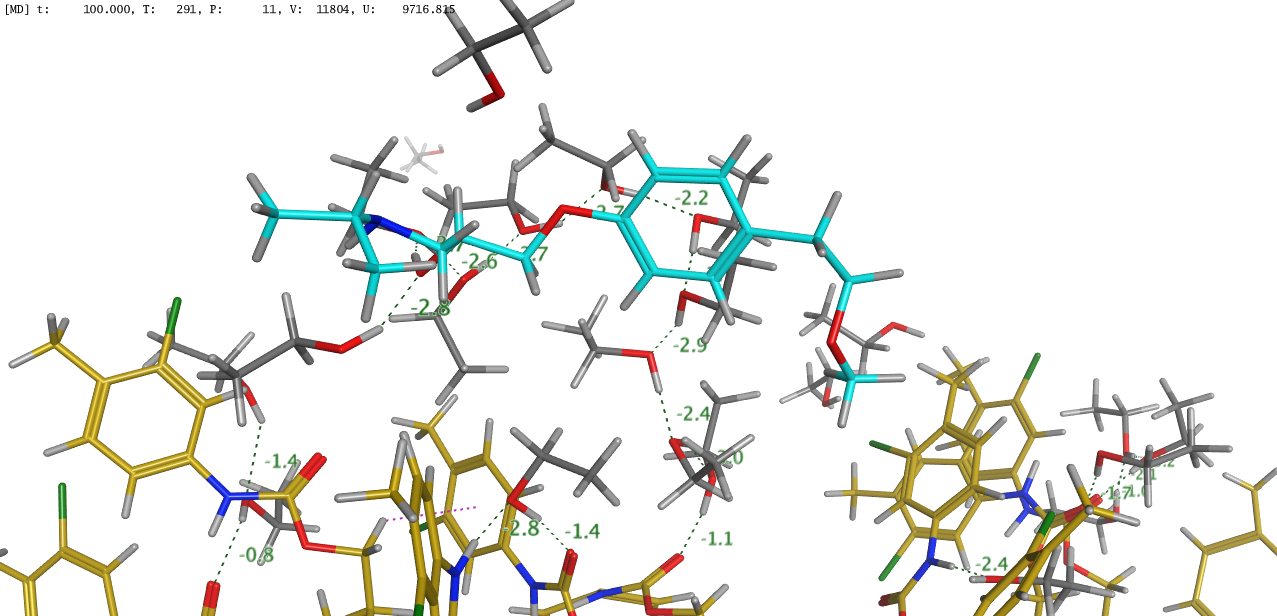


**Fig. S29:** Binding interactions of S-metoprolol with CSP throughout dynamic simulation at 100 ps.


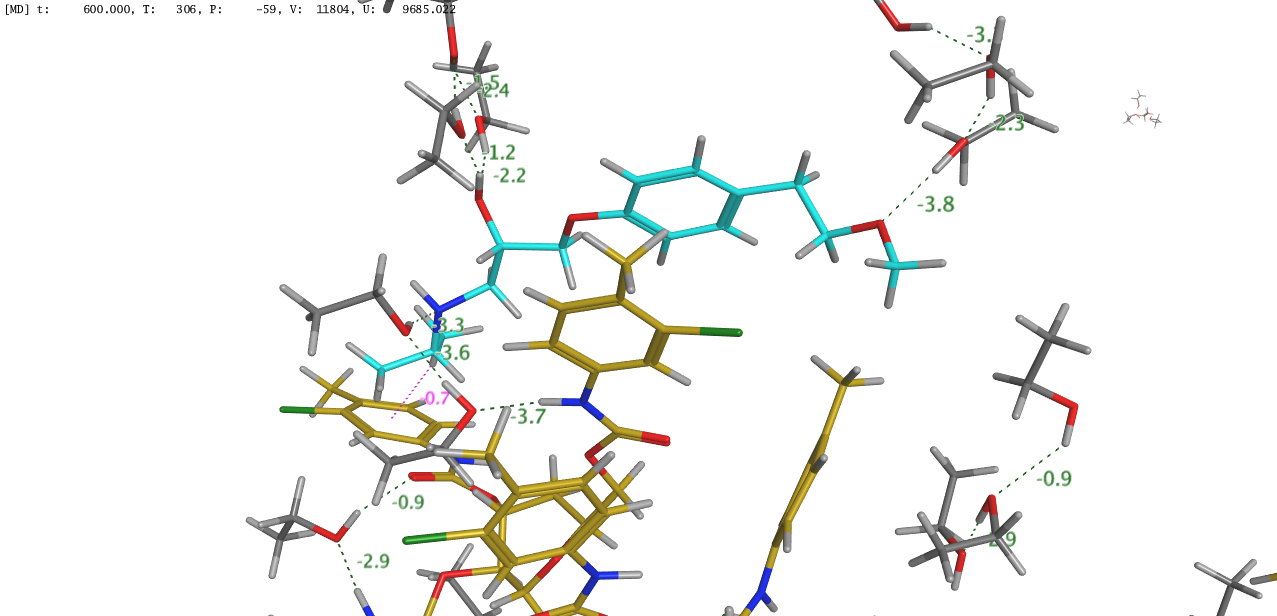


**Fig. S30:** Binding interactions of S-metoprolol with CSP throughout dynamic simulation at 600 ps.
